# Characteristics of the nasal mucosa of commercial pigs during normal development

**DOI:** 10.1186/s13567-023-01164-y

**Published:** 2023-04-24

**Authors:** Yuchen Li, Chengjie Yang, Yuqi Jiang, Xiuyu Wang, Chen Yuan, Jiaxin Qi, Qian Yang

**Affiliations:** MOE Joint International Research Laboratory of Animal Health and Food Safety, College of Veterinary Medicine, Nanjing Agricultural University, Nanjing, Jiangsu China

**Keywords:** Commercial pig, nasal cavity, mucosa barrier structure and composition, microflora

## Abstract

The nasal mucosa is constantly exposed to inhaled pathogens and is the first defence against respiratory infections. Here, we investigated the structural and compositional characteristics of the nasal mucosa of commercial pigs at various growth stages. The epithelial thickness, number of capillaries, and secretion function of the nasal mucosa dramatically increased with age; however, underlying lymphoid follicles in the respiratory region were rarely observed across the growth stages. The nasal mucosa was explored at the epithelial, immunological, and biological (commensal microbiota) barriers. In the epithelial barrier, the proliferative capacity of the nasal epithelia and the expression of tight junction proteins were high after birth; however, they decreased significantly during the suckling stage and increased again during the weaning stage. In the immunological barrier, most pattern recognition receptors were expressed at very low levels in neonatal piglets, and the innate immune cell distribution was lower. During the suckling stage, increased expression of Toll-like receptor (TLR) 2 and TLR4 was observed; however, TLR3 expression decreased. TLR expression and innate immune cell quantity significantly increased from the weaning to the finishing stage. In the biological barrier, *Firmicutes*, *Actinobacteria*, *Proteobacteria*, and *Bacteroidetes* comprised the dominant phyla in neonatal piglets. A dramatic decrease in nasal microbial diversity was observed during the suckling stage, accompanied by an increase in potentially pathogenic bacteria. *Proteobacteria*, *Bacteroidetes*, and *Firmicutes* were identified as the core phyla of the nasal microbiota; among these, the three dominant genera, *Actinobacter*, *Moraxella*, and *Bergerella*, may be opportunistic pathogens in the respiratory tract. These characteristics comprise an essential reference for respiratory infection prevention at large-scale pig farms.

**Supplementary Information:**

The online version contains supplementary material available at 10.1186/s13567-023-01164-y.

## Introduction

With the large-scale expansion of swine production, respiratory diseases caused by pathogenic microorganisms such as bacteria and viruses have become increasingly severe, leading to substantial losses for the global swine industry [1, 2]. The nasal cavity is an entry route into the respiratory tract continuously exposed to external threats [3, 4]. The mucosal layer that lines the nasal cavity is the first defence against the invasion of potentially pathogenic microorganisms. Therefore, strengthening the barrier of the nasal mucosal tissue could be the most effective way to prevent or control porcine respiratory disease [5, 6]. Therefore, a better understanding of the nasal mucosa’s structural and functional characteristics is essential to achieving this goal.

Our previous studies revealed the histological and immunocytic characteristics of the nasal mucosa of Bama minipigs [7]. Similar to humans and rodents, the nasal cavity of Bama minipigs is comprised of the following three regions with different functional characteristics: the vestibular region, the respiratory region, and the olfactory region. Although lymphoid tissues are randomly distributed in the nasal cavity of Bama minipigs, the distribution of multiple innate immune cells (including CD3^+^ T cells, immunoglobulin A [IgA]^+^ cells, and dendritic cells [DCs]) increased distally in the nasal cavity. The Bama miniature pig is a unique small-scale breed from southern China that has been increasingly developed as an animal model for medical studies [8]. However, because of their significant differences in body size (smaller) and growth performance (slower), studies on Bama minipigs have provided results that are of limited value for understanding the structure and function of the nasal mucosa of commercial pigs. Furthermore, related studies of commercial pigs are lacking. Therefore, we sought to explore the structural and functional characteristics of the nasal mucosa of commercial pigs based on the findings of studies involving Bama minipigs.

The composition of the upper respiratory tract mucosa in the nasal cavity is similar to that of the intestinal mucosal barriers. It has a multi-layered structure, including epithelial mechanical, immunological, and biological barriers [9]. The mechanical barrier is the structural basis and primary component of the nasal mucosa, mainly composed of nasal epithelial cells, and has tight connections between adjacent epithelial cells [10]. Among nasal epithelial cells, mucus-secreting goblet cells form a thick protective mucus layer that covers the nasal epithelium and can act as an essential addition to the epithelial mechanical barrier [11]. Strategically located in the subepithelial compartment, innate immune cells in the nasal lamina propria are crucial for immune surveillance at mucosal sites. The distribution of macrophages, DCs, B cells, and intraepithelial T cells (IETs) in porcine nasal mucosa has been identified [12, 13]. Mucosal resident innate immune cells exert a rapid immune response and form a powerful immune barrier in the nasal mucosa. The function of the nasal immune barrier is triggered and orchestrated by a variety of pattern recognition receptors expressed by nasal epithelial cells and innate immune cells [14]. Mucosal sites, such as the intestine, oral cavity, nasopharynx, and vagina, have associated commensal microbiota [15].

Commensal microbiota is required to maintain or repair the barrier functions of the mucosal tissue. Studies have shown that the mucosal commensal microbiota forms a biological barrier at multiple levels and plays an essential role in inhibiting pathogenic colonization and promoting nasal mucosal barrier stability [16]. Under normal circumstances, both the microbiota and their metabolites protect this biological barrier function [17]. Together, these functional barriers are the basis of the protective role of the mucosal immune barrier. Therefore, an in-depth understanding of their composition and structural features is vital in investigating the barrier function of the nasal mucosa.

The structure, composition, and function of mucosal tissue vary greatly at different ages [18, 19]. These differences may lead to dramatically different mucosal barrier functions in pigs at different growth stages and influence their susceptibility and immune response to mucosal pathogenic infections. For example, neonatal piglets are more susceptible to intestinal infections caused by porcine epidemic diarrhoea virus, porcine delta coronavirus, and rotavirus because of their underdeveloped intestinal mucosal barrier [20]. Clinically, the primary strategy for preventing diarrhoea in neonatal pigs is using a maternal vaccine to boost passively transferred protective antibodies [21]; considering the similar age-related susceptibility of pigs to respiratory pathogen infections [22], the nasal mucosa characteristics that differ according to age warrant further investigation.

We conducted a systematic study of the nasal cavity of commercial pigs at different growth stages. The barrier characteristics of the nasal mucosa were explored by studying the structural and compositional features of the epithelial, mechanical, immunological, and biological barriers. Comprehensive knowledge of the nasal mucosa will provide a valuable reference for developing effective mucosal immunological strategies that will help block the route of entry for respiratory pathogens in pigs.

## Materials and methods

### Antibodies and reagents

ZO-1 monoclonal antibody (1:200, Z01-1A12, Invitrogen, USA) was used to determine the expression of the tight junction protein zonula occludens-1 (ZO-1). To observe and analyse nasal immune cells, goat anti-pig IgA (1:100; A100102P; Bethyl, USA), rabbit anti-pig CD3 (1:200; ab16669; Abcam, USA), and rabbit anti-pig CD163 polyclonal antibodies (1:1000; 16,646–1-AP, Proteintech, China) were used. Other antibodies included anti-pig proliferating cell nuclear antigen (PCNA) antibody (1:200; ab29; Abcam, USA) and secondary antibodies used for indirect immunofluorescence assays, such as AlexaFluor488-conjugated donkey anti-mouse IgG1 (1:200; A-21202; Invitrogen, USA). The SABC-POD (rabbit or goat IgG) kit (Boster Bioengineering, China) was also used. Periodic-acid-Schiff (PAS) staining was performed using a PAS kit (Beijing Leagene, China) according to the manufacturer’s recommendations.

### Animals

Neonatal (age, 0 days; weight, 1.1–1.3 kg), suckling (age, 7 days; weight, 2.4–4 kg), weaner (age, 1 month; weight, 8–10 kg), grower (age, 2 months; weight, 15–20 kg), and finisher (age, 6 months; weight, 40–50 kg) Duroc × Landrace × Yorkshire pigs were obtained from a swine herd at the Jiangsu Academy of Agricultural Science. The swineherd was seronegative for antibodies against porcine reproductive and respiratory syndrome virus, porcine pseudorabies virus, porcine epidemic diarrhoea virus, and porcine circovirus type 2. All pigs had free access to water and food at all times.

The neonatal piglets were sacrificed 6 h after birth. The other pigs were transported to the experimental animal centre of Nanjing Agricultural University, China, where they were raised under highly sanitary conditions for at least 48 h before the experiment to reduce the stress response and avoid potential pathogenic infection. The pigs had free access to water and food at all times. The suckling piglets were fed milk every three hours throughout the experiment and a corn‒soybean meal-based diet that met or exceeded the nutrient and energy requirements for their size (NRC, 2012). All animal studies were approved by the Institutional Animal Care and Use Committee of Nanjing Agricultural University (SYXK-2017–0027) and followed the National Institutes of Health guidelines for the performance of animal experiments.

### Collection and preparation of nasal samples

**Figure 1:**
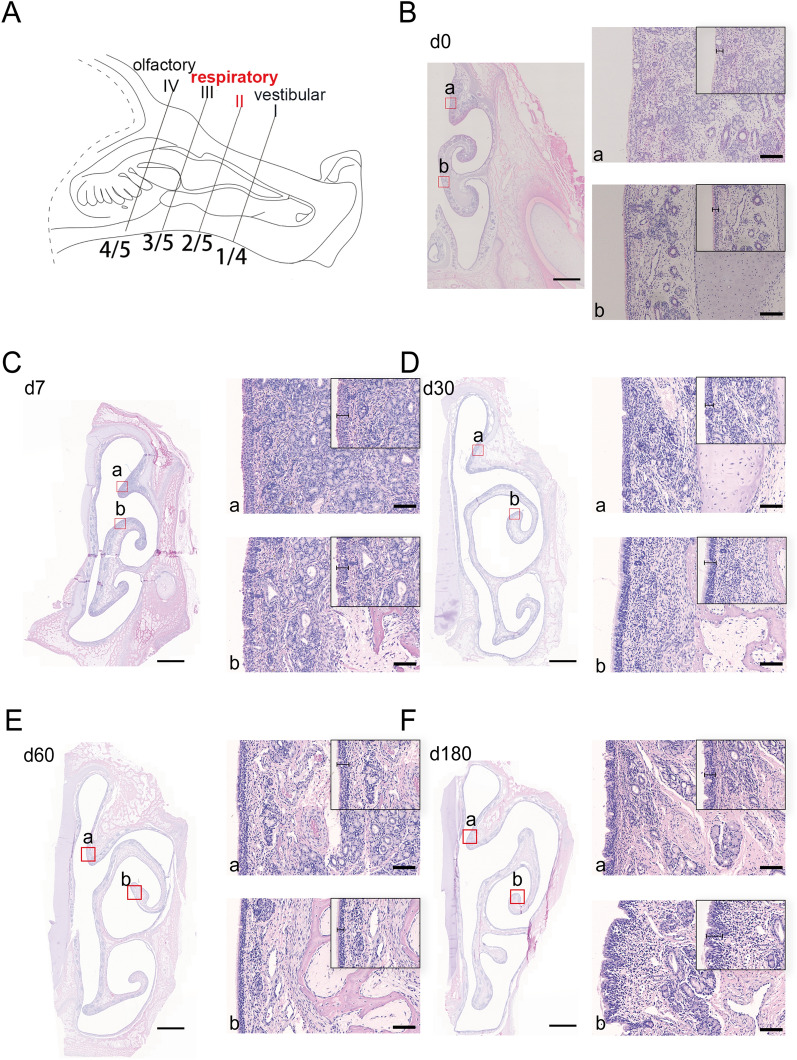
**HE staining of the anterior part of the nasal respiratory region in pigs of different ages.** **A** Diagrams of cross-section II (corresponding to the anterior part of the respiratory region) of the pig nasal cavity. **B**–**F** Representative images of HE-stained nasal anterior respiratory regions from pigs at different ages, including 0 days old (**B**), 7 days old (**C**), 30 days old (**D**), 60 days old (**E**) and 180 days old (**F**). The red frame in each figure indicates the superior nasal concha (**a**) and inferior nasal concha (**b**), and magnified images of the corresponding region are shown on the right of the figure. Scale bars: **B**–**F** 2 mm; (**a**, **b**) 50 μm.

Five pigs from each growth stage were randomly selected and euthanized with pentobarbital sodium (100 mg/kg). Subsequently, nasal swabs and nasal cavity samples were immediately collected. The pigs were decapitated to obtain an intact nasal cavity, and the lower jaw, skin, adipose tissue, and muscle tissue were removed. The nasal cavity was then fixed with 4% paraformaldehyde for 48 h at 25 ℃. After fixation, the nasal cavity was divided into four cross-sections (I–IV) according to the ratios of 1/4, 2/5, 3/5, and 4/5 (as depicted in the pattern diagrams presented in Figure 1A). Cross-section I corresponded to the vestibular region. Cross-sections II and III corresponded to the respiratory region. Cross-section IV corresponded to the olfactory region. The fixed tissues of the nasal cavity from the weaner, grower and finishing pigs were immersed in 10% EDTA solution for 2 weeks for decalcification for 2 weeks; the decalcified solution was changed every three days.

The calcium oxalate test was used to determine the decalcification endpoint. The nasal cavity was considered decalcified when the addition of saturated ammonium oxalate failed to produce a precipitate. Blocks from parts of the nose were trimmed to fit the slides and dehydrated in sequential gradient alcohol (75%, 85%, 95%, and 100%) and xylene baths. After dehydration, the tissues were embedded in paraffin and serially sliced into 6-μm-thick sections. Subsequently, the sections from four cross-sections (I, II, III, and IV) were selected for haematoxylin and eosin (HE) or PAS staining and observed using a BX51 digital camera system (Olympus Corporation, Tokyo, Japan).

### Histological analysis

For histological evaluation, HE staining was performed on the sections using a HE staining kit (Solarbio, Beijing, China). The number of glands in the lamina propria was counted in five randomly selected visual fields (10×) of the five individual sections, summarized in a column plot. The number of capillaries and glands in the nasal mucosa were counted in five randomly selected visual fields (10×) of the five individual sections, summarized in a column plot. The epithelial thickness of the nasal mucosa was measured using ImageJ software, and five visual fields (40×) were randomly selected from the five individual sections. All the measurements, summarized in the column plot, are provided as the average epithelial thickness per group. The turbinate area in the nasal respiratory was measured using ImageJ software; the results were obtained from five individual sections. The goblet cells in the nasal mucosa were stained using a periodic acid-Schiff (PAS) staining kit (Solaibio, China). The number of PAS-positive cells (goblet cells) was counted in five visual fields (10 ×) of the five individual sections. The bar histogram displays the quantification of PAS-positive cells in each group.

### Immunohistochemistry

Paraffin sections were dewaxed and rehydrated by incubation with xylene and descending ethanol concentrations. To achieve antigen retrieval, the fixed sections were placed in citrate buffer (pH 6; 90–95 °C) for 30 min. After eliminating endogenous peroxidase activity (0.5% H_2_O_2_), the sections were rehydrated in phosphate-buffered saline, permeabilized with 0.5% Triton/phosphate-buffered saline, and blocked with 5% bovine serum albumin for two hours at 25 °C. The sections were then incubated with primary antibodies (mouse anti-ZO-1 antibody, rabbit anti-CD3 antibody, goat anti-IgA antibody, rabbit anti-CD163 antibody) overnight at 4 ℃ in a humidified chamber. The SABC-POD kit was used for signal amplification and visualization. Sections in which the primary antibodies were omitted were used as negative controls. The sections were visualized under a light microscope (Olympus CX23; Olympus Corporation) at 40 × or 10 × magnification. Different fields (40 × or 10 × ; *n* = 5 for each pig tissue) were counted during the statistical analysis.

### Immunofluorescence and confocal microscopy

To assess proliferation, tissue sections were rinsed and subjected to antigen repair, as described previously. After rinsing in phosphate-buffered saline, samples were treated with 5% bovine serum albumin for two hours at 25 ℃ and incubated with an anti-pig PCNA antibody. Anti-mouse Alexa 488 secondary antibodies were used to visualize primary antibody labelling. The negative control slides were treated identically, except for removing the primary antibodies. The cell nuclei were stained by incubation with diamidino-2-phenylindole for 5 min and observed under a confocal laser microscope (LSM-710; Zeiss, Oberkochen, Germany).

### RNA isolation and quantitative real-time polymerase chain reaction

Total RNA was extracted from the nasal mucosa using TRIzol reagent (Takara Biotechnology (Dalian, China) Co., Ltd.) according to the manufacturer’s instructions. cDNA was generated by reverse transcription using HiScript™ QRT SuperMix (Vazyme, China) according to the manufacturer’s instructions. Target gene transcription was performed using quantitative real-time polymerase chain reaction (PCR) with a SYBR Green qPCR kit (Takara Biotechnology (Dalian, China) Co., Ltd.) and analysed using the double standard curve method. All primers used for quantitative real-time PCR are listed in Additional file 1. The gene expression levels were normalized to those of glyceraldehyde 3-phosphate dehydrogenase. Relative levels of cytokine RNA were calculated using the 2^−ΔΔCT^ method.

### DNA extraction and 16S rDNA sequencing

According to the manufacturer’s protocols, DNA was extracted from nasal swab fluid using the HiPure Soil DNA kit (Magen, Guangzhou, China). The quality of the genomic DNA was determined using a Thermo NanoDrop 2000 ultraviolet microspectrophotometer (Thermo Fisher Scientific Inc., Waltham, MA, USA) and 1% agarose gel. The V3-V4 hypervariable regions of the bacterial 16S rDNA gene were amplified using universal 16S rDNA primers (forward: 5′-GTGCCAGCMGCCGCGG-TAA-3′; reverse: 5′-GGACTACHVGGGTWTCTAAT-3′) and tagged with an Illumina adaptor sequence at the 5′ end. Using diluted genomic DNA as a template, PCR was performed using the KAPA HiFi HotStart ReadyMix PCR kit high-fidelity enzyme (Kapa Biosystems Inc., Boston, MA, USA). The PCR product was detected by 2% agarose gel electrophoresis and recovered by gelatinization using the AxyPrep DNA gel recovery kit (Axygen Scientific Co., Union City, CA, USA). After recovery, library quality checks were performed using a NanoDrop 2000 ultraviolet spectrophotometer (Thermo Fisher Scientific Inc.) and 2% agarose gel electrophoresis. The PCR products were sequenced using Illumina HiSeq PE250 (Illumina, San Diego, CA, USA).

### Nasal microbiota analysis

Sequences were clustered into operational taxonomic units (OTUs) using USEARCH (v7.0.1090) with 97% identity. The relative abundance of OTUs was calculated and classified into various taxonomic ranks (phylum, order, class, family, genus, and species). Based on the OTUs and species annotation, the sample species complexity and species differences among the groups were analysed. The OTU Venn diagram was constructed using R statistical software (v3.1.1; R Foundation for Statistical Computing, Vienna, Austria) for OTU overlap in different groups. The α-diversity (including Chao1 value, ACE value, Shannon index, and Simpson index) and β-diversity of the sample were evaluated based on the OTU results. The microbiota composition was analysed at different biological classification levels (from phylum to genus). A heatmap was constructed to identify specific microbiota types. Kyoto Encyclopedia of Genes and Genomes pathway analysis of OTUs/amplicon sequence variants was performed using Tax4Fun, and the microbiome phenotypes of bacteria were classified using BugBase. The functional differences among groups were evaluated using Welch’s t test, the Wilcoxon rank test, and the Kruskal–Wallis H test with R statistical software. Sequencing was performed by Gene Denovo Biotechnology Co., Ltd. (Guangzhou, China). Sequence data were deposited in the National Center for Biotechnology Information Sequence Read Archive under accession number PRJNA815911.

### Statistical analysis

The results are presented as the mean ± standard deviation and were analysed using SPSS v.17.0 (SPSS Inc., Chicago, IL, USA). A one-way analysis of variance was performed to compare significant differences among multiple groups. A significant difference was reported when *P* < 0.05. A highly significant difference was reported when *P* < 0.01. All results were obtained from at least three independent experiments unless otherwise stated.

## Results

### Histological characteristics of the porcine nasal cavity at various growth stages

Nasal tissue samples were collected from pigs at all growth stages (neonatal, 0 days; suckling, seven days; weaning, 30 days; fattening, 60 days; and finishing, 180 days). Subsequently, four cross-sections of the porcine nasal cavity were selected to undergo an examination of the histological structure. Cross-section I corresponded to the vestibular region. Cross-sections II and III corresponded to the respiratory region. Cross-section IV corresponded to the olfactory region. The vestibular region of the nasal cavity was entirely covered by stratified squamous epithelia, which provide structural and functional defence against invading pathogens (Additional file 2). With increasing age, the stratified squamous epithelia and their transitional layer (towards the pseudostratified columnar ciliated epithelium) were both markedly thickened (Additional file 3). Interestingly, the finishing pigs displayed extensions of the epithelial rete pegs into the underlying connective tissue (Additional file 2). In addition, compared with neonatal and suckling piglets, fattening and finishing pigs had more capillaries and glands in lamina propria cross-Section I (Additional file 3).

The position of the anterior part of the respiratory region was marked in the pattern diagrams of the nasal cavity (Figure 1A). In the anterior part of the respiratory region, conch-shaped, curved turbinate rolls of the inferior turbinate were formed and occupied a relatively small portion of the nasal passage. Although the covered epithelium gradually transitioned to pseudostratified columnar ciliated epithelia in this region, the epithelial transition characteristics differed among age groups (Figures 1B–F). At 30 days of age or older, the dorsal spiral of the superior or inferior nasal concha was entirely covered by pseudostratified columnar ciliated epithelia (Figures 1D–F); however, a similar part was covered by simple columnar epithelium in neonatal and suckling piglets (Figures 1B and C). There were significantly more glands in the inferior turbinate than in the superior turbinate. With increased age, more mucous glands and capillary vessels were observed in the mucosal lamina propria of the superior and inferior nasal concha (Additional file 3). The thickness of the covered epithelial layer increased with age; however, a slight decrease (*p* < 0.05) was observed at the age of 180 days (Additional file 3).

**Figure 2:**
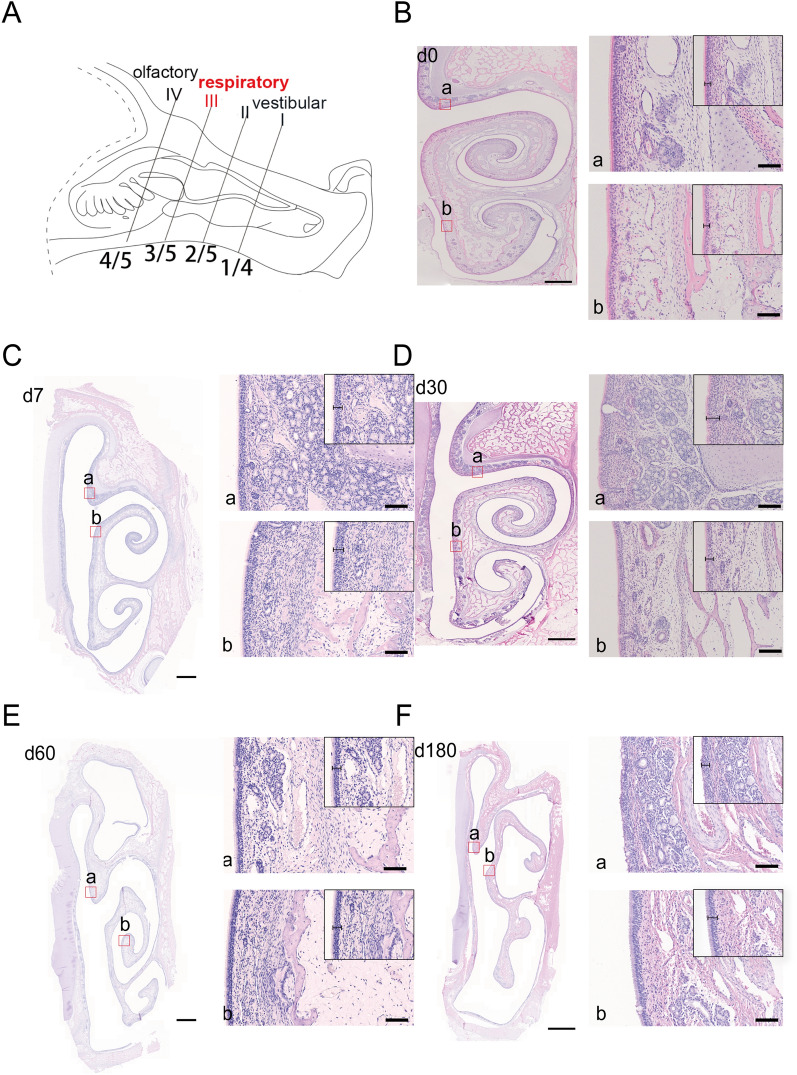
**HE staining of the rear part of the nasal respiratory region in pigs of different ages.** **A** Diagrams of cross-section III (corresponding to the rear of the respiratory region) of the pig nasal cavity. **B**–**F** Representative images of HE-stained rear part of the nasal respiratory region from pigs at different ages, including 0 days old (**B**), 7 days old (**C**), 30 days old (**D**), 60 days old (**E**), and 180 days old (**F**). The red frame in each figure indicates the superior nasal concha (**a**) and inferior nasal concha (**b**); magnified images of the corresponding region are shown on the right of the figure. Scale bars: **B**–**F** 2 mm; (**a**, **b**) 50 μm.

At the rear part of the respiratory region (Figure 2A), the spiral-shaped turbinate curved further medially and was significantly enlarged to the point where it occupied most of the space in the nasal passages, thus dividing them into the upper, middle, lower, and general nasal passages (Figures 2B–F). The covered epithelium in the superior or inferior nasal concha was transformed into pseudostratified columnar ciliated epithelia in all age groups, and the epithelial thickness increased with age (Additional file 3). In contrast to the anterior part of the respiratory region, there were significantly fewer glands in the inferior turbinate of the retral respiratory region than in the superior turbinate (Additional file 3). Notably, only a reduced number of lymphoid follicles were found in the lamina propria of the respiratory region of finishing pigs. At the same time, lymphoid tissue could hardly be observed in commercial pigs of other ages (Additional file 4).

Finally, the nasal cavity of the olfactory region preserved the middle nasal concha and nasopharyngeal meatus (Additional file 5). The olfactory epithelium is a pseudostratified epithelium between the nasal septum and lateral walls of the nasal cavities. The thickness of the olfactory epithelium covering the middle turbinate and nasal septum also increased with age. There was a similar pattern for the number of underlying capillaries and glands (Additional file 3).

### Characteristics of the physical barrier of the nasal cavity at different growth stages

**Figure 3:**
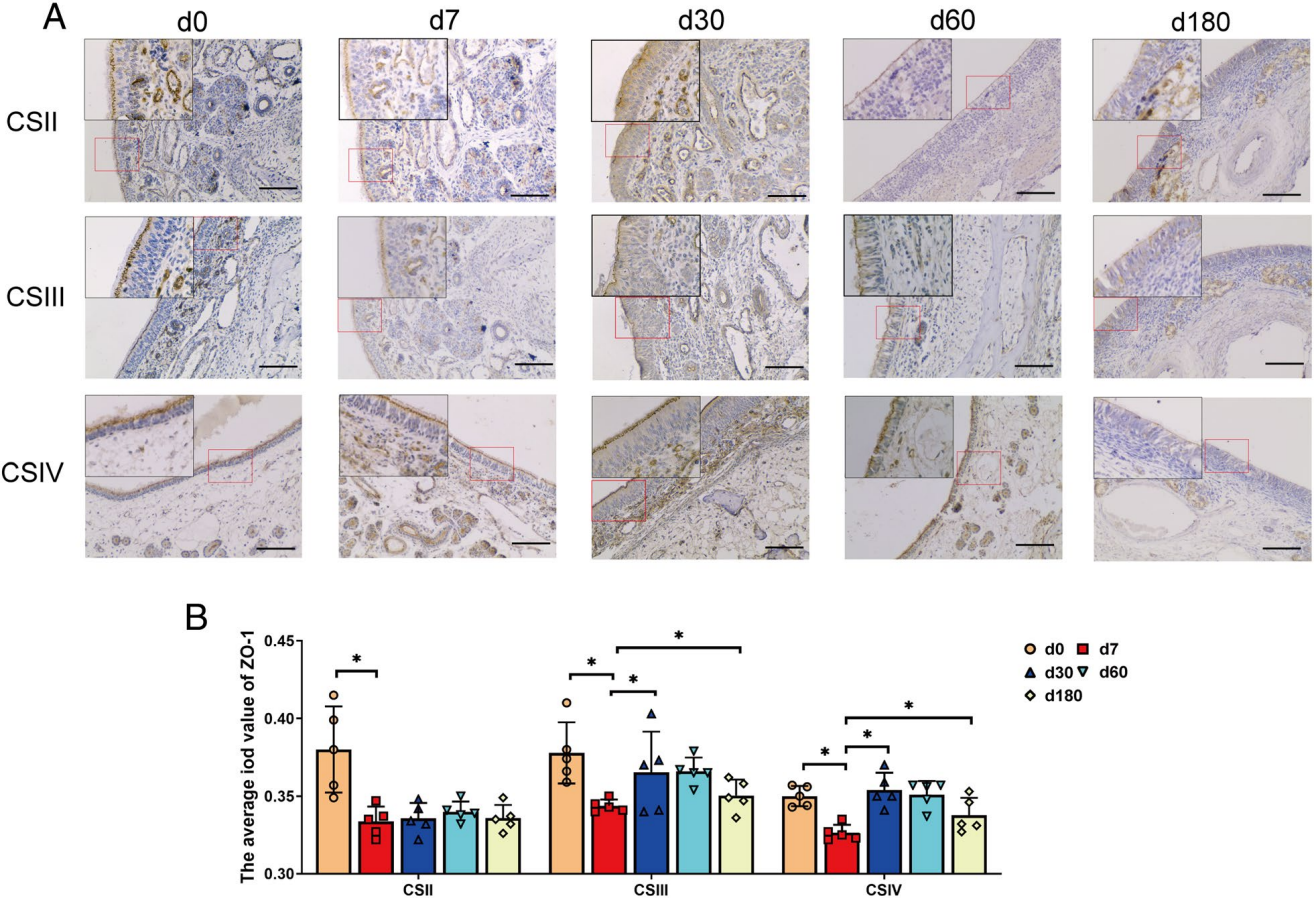
**The expression of ZO-1 in the nasal epithelium of pigs at different ages.** **A** Immunohistochemical staining of the tight junction protein ZO-1 in the nasal epithelium. CSII represented the anterior part of the respiratory region, CSIII represented the rear part of the respiratory region, and CSIV represented the olfactory region. Scale bars: 100 μm **B** Bar chart summarizing the statistical analysis of ZO-1 staining results in each group, counted in five random fields (×40) from four cross-sections. All data shown are the mean ± SD from three independent experiments. Statistical significance was obtained using one-way ANOVA. NS no significance, *P < 0.05, **P < 0.01.

The nasal epithelium provides a basic physical barrier that helps maintain the stability of the mucosal barrier. Tight junction proteins are located in the apicolateral domains of epithelial cells. They seal the epithelial layer and comprise the first line of defence against invading microorganisms [23]. To visualize the changes in tight junctions throughout the growth stages, the expression level of the tight junction protein ZO-1 was evaluated in the nasal epithelium (Figure 3). After birth, high expression in the olfactory and respiratory regions of the nasal cavity was observed, followed by a rapid decrease in expression during the suckling period. Subsequently, the expression of ZO-1 exhibited distinct trends in different parts of the nasal cavity. In the anterior part of the respiratory region, its expression was low until the finishing stage; however, it increased in the rear of the respiratory and olfactory regions at the weaning stage.

**Figure 4:**
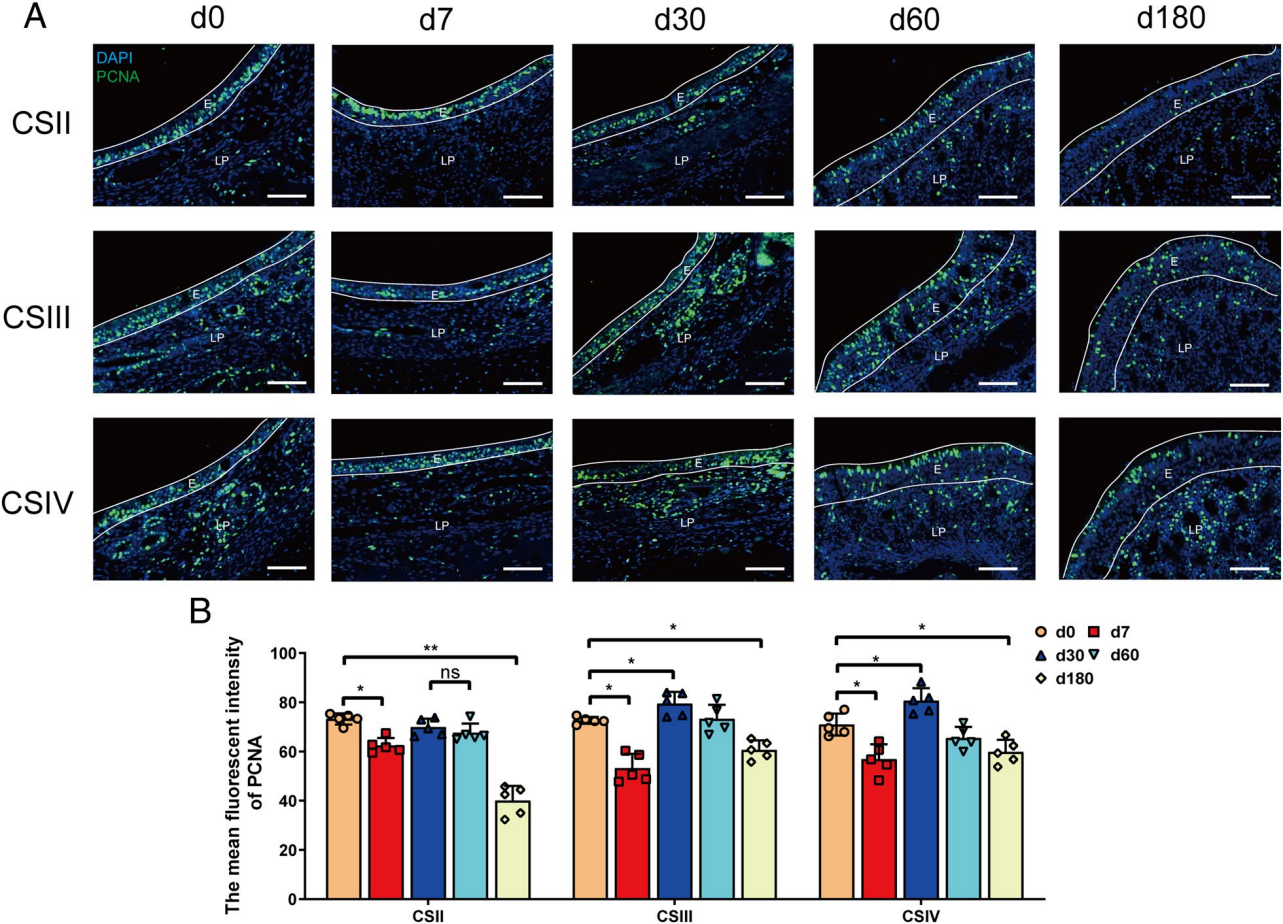
**The expression of PCNA in the nasal epithelium of pigs at different ages.** **A** Immunofluorescence assessment of PCNA expression in the nasal epithelium. CSII represented the anterior part of the respiratory region, CSIII represented the rear part of the respiratory region, and CSIV represented the olfactory region. Scale bars: 50 μm **B** Statistical results of PCNA expression. The mean fluorescent intensity of PCNA-positive cells (green) in different parts of the cavity was counted in five random fields (×20) from the four cross-sections. All data shown are the mean ± SD from three independent experiments. Statistical significance was obtained using one-way ANOVA. NS no significance, *P < 0.05, **P < 0.01.

Epithelial regeneration is critical for maintaining physical barrier function after mucosal injury. PCNA expression is a good indicator of epithelial cell proliferation [24]. A similar expression pattern of PCNA in the nasal epithelium was identified in the respiratory and olfactory regions (Figure 4). After birth, a high level of PCNA expression was observed; however, this decreased significantly during the suckling stage. After the suckling stage, the expression of PCNA increased again and peaked; its expression level then decreased at the finishing stage, with the most significant decline detected in the anterior part of the nasal respiratory region.

**Figure 5:**
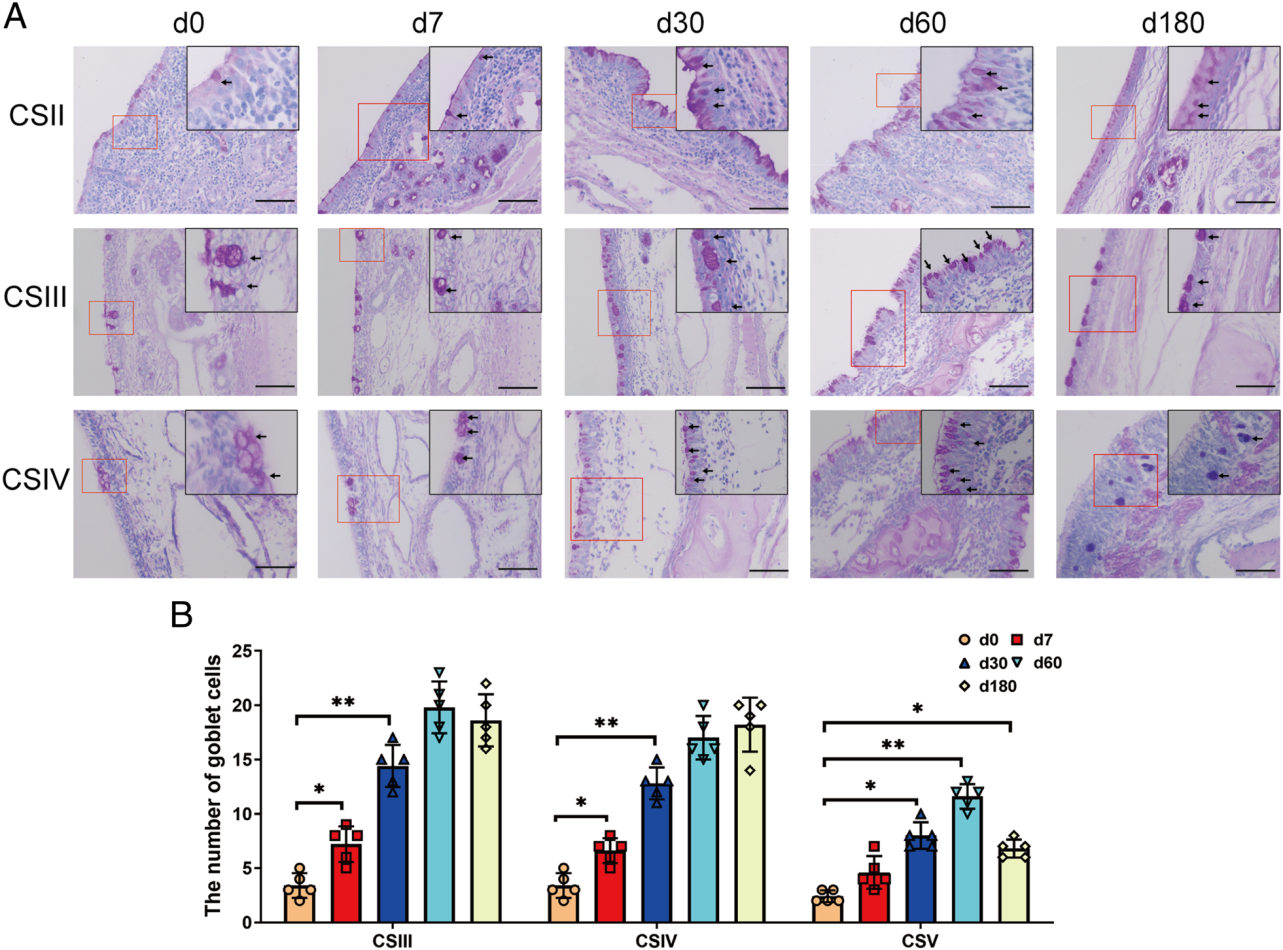
**The distribution of goblet cells in the nasal epithelium of pigs at different ages.** **A** Representative images of PAS staining in the nasal epithelium. CSII represented the anterior part of the respiratory region, CSIII represented the rear part of the respiratory region, and CSIV represented the olfactory region. Scale bars: 100 μm. **B** Quantification analysis of PAS staining. The number of PAS-positive cells (pink) in different parts of the cavity was counted in five random fields (×10) from the four cross-sections. All data shown are the mean ± SD from three independent experiments. Statistical significance was obtained using one-way ANOVA. NS no significance, *P < 0.05, **P < 0.01.

Goblet cells are located within the epithelial layer and produce mucus to form a protective layer covering the nasal epithelium [25]. PAS staining revealed a greater number of epithelial goblet cells in the respiratory region than that in the olfactory region (Figure 5). Specifically, ageing was associated with a significant increase in the number of goblet cells in the respiratory and olfactory regions. However, the number of goblet cells in the olfactory region decreased significantly at the finishing stage.

### Immune barrier characteristics of the nasal cavity at different growth stages

**Figure 6:**
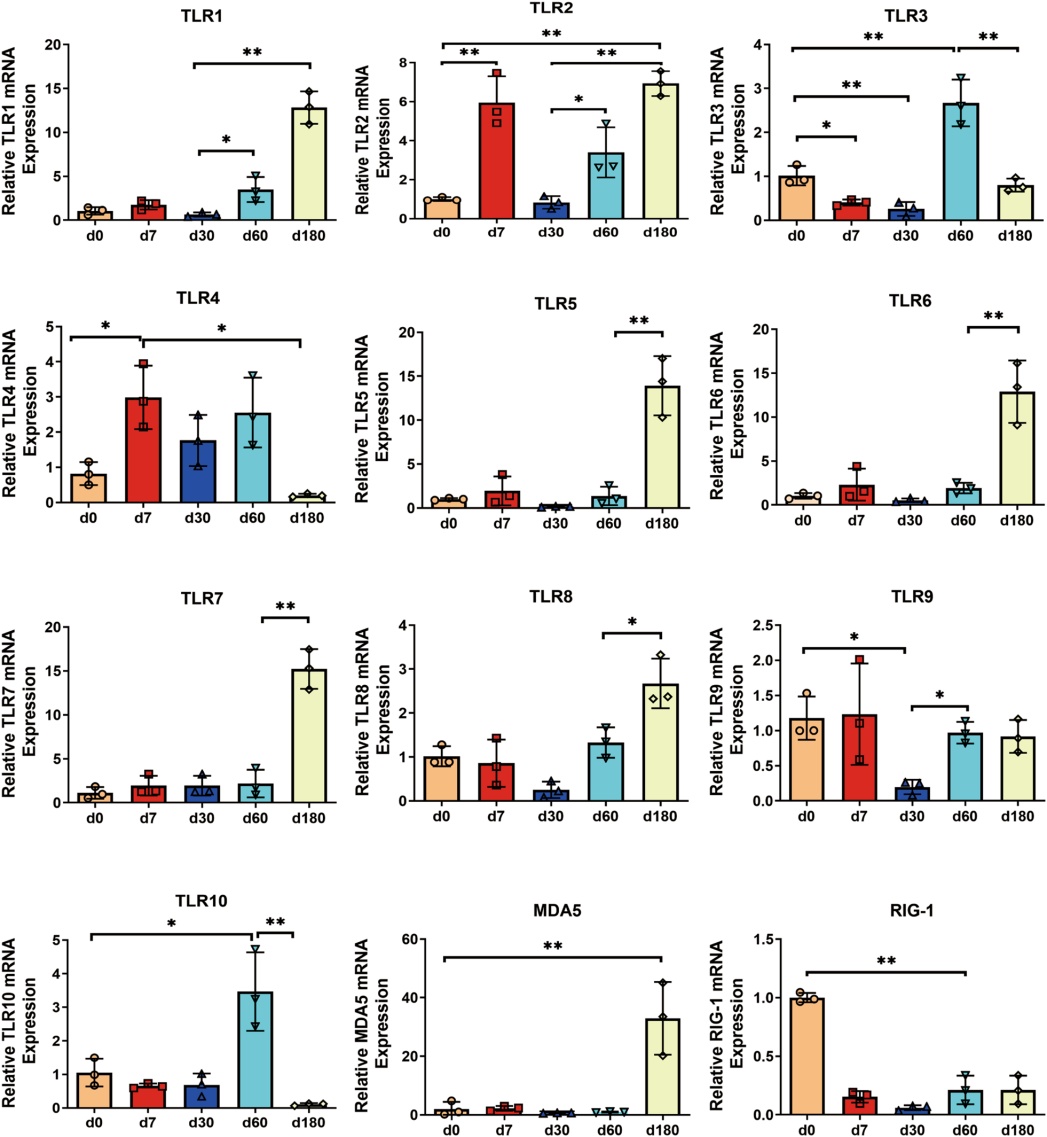
**The expression of pattern recognition receptors (PRRs) in the nasal mucosa of pigs at different ages.** The transcription of Toll-like receptors (TLRs) and RIG-I-like receptors (RLRs) in nasal mucosa was detected via RT‒qPCR. TLRs include cell surface receptors (TLR1, TLR-2, TLR-4, TLR-5, TLR-6, and TLR-10) and intracellular receptors (TLR-3, TLR-7, TLR-8, TLR-9). RLRs include MDA5 and Rig-I. All data shown are the mean ± SD from three independent experiments. Statistical significance was obtained using one-way ANOVA. NS no significance, *P < 0.05, **P < 0.01.

Pattern recognition by receptors is critical in immune surveillance at the mucosal site [26]. The mRNA expression of Toll-like receptors (TLRs) and that of the retinoic acid-inducible gene (RIG), the most critical recognition molecules, were determined in the nasal mucosa (Figure 6). Among the TLR family members, the expression of TLR2 and TLR4 was significantly upregulated during the suckling stage, whereas TLR3 expression was suppressed. Weaning was associated with the inhibition of TLR2 and TLR9 expression, which increased again during the fattening stage. From birth to the fattening stage, the expression of TLR5, TLR6, TLR7, and TLR8 remained stable; however, the expression of these molecules was significantly upregulated during the finishing stage. Similar trends were reflected in the expression of TLR1; however, its expression increased at the fattening stage. The expression of TLR10 also increased at the fattening stage; however, it decreased again to relatively low levels at the finishing stage (lower than the expression level detected at birth). MDA5 and RIG-I, which are RIG-like receptors, exhibited opposite expression trends throughout the growth stages. The expression of MDA5 maintained a low level from birth to the fattening stage, and it was significantly upregulated at the finishing stage. After birth, high RIG-I expression was detected; however, it decreased rapidly at the suckling stage and remained stable until the finishing stage.

**Figure 7:**
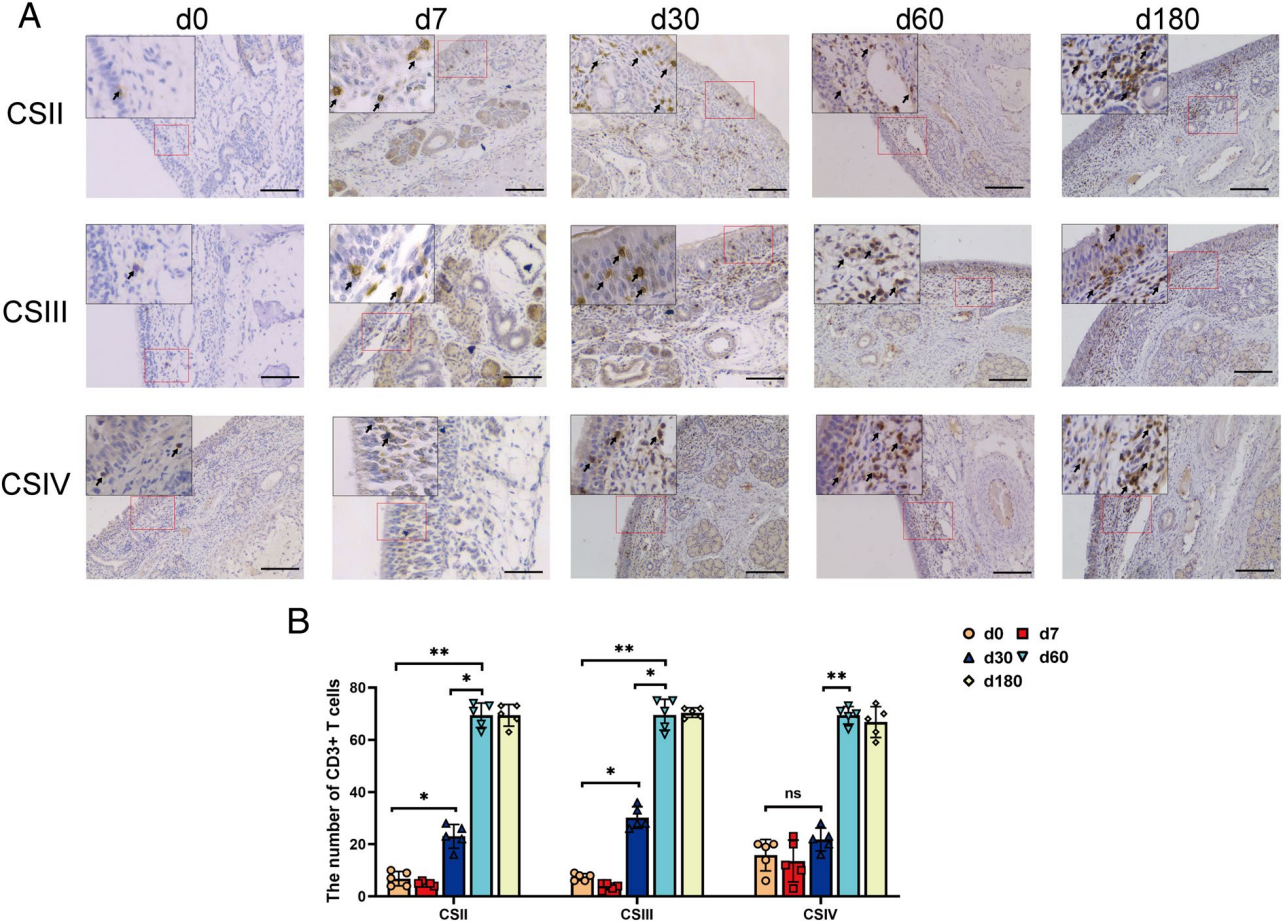
**The location of CD3**^**+**^** T cells within the nasal epithelium of pigs at different growth stages.** **A** IHC staining for CD3^+^ T cells in the nasal epithelium. T-cell-specific CD3 protein was stained brown. CSII represented the anterior part of the respiratory region, CSIII represented the rear part of the respiratory region, and CSIV represented the olfactory region. Scale bars: 100 μm. **B** Quantification analysis of CD3 + T cells. The number of CD3-positive cells in different parts of the nasal cavity was counted in five random fields (×10) from the four cross-sections. All data shown are the mean ± SD from three independent experiments. Statistical significance was obtained using one-way ANOVA. NS no significance, *P < 0.05, **P < 0.01.

Located within nasal epithelial cells, IETs serve as immune guardians at the front of the mucosal barrier [27]. Overall, IETs increased substantially in the respiratory and olfactory regions throughout the growth stages. Similar distribution patterns were identified in both areas (Figure 7). Fattening and older pigs exhibited the largest IET distribution. At the same time, few IETs were detected in neonatal and suckling piglets. At the weaning stage, the number of IETs significantly increased in the respiratory region; however, it did not substantially change in the olfactory region.

**Figure 8:**
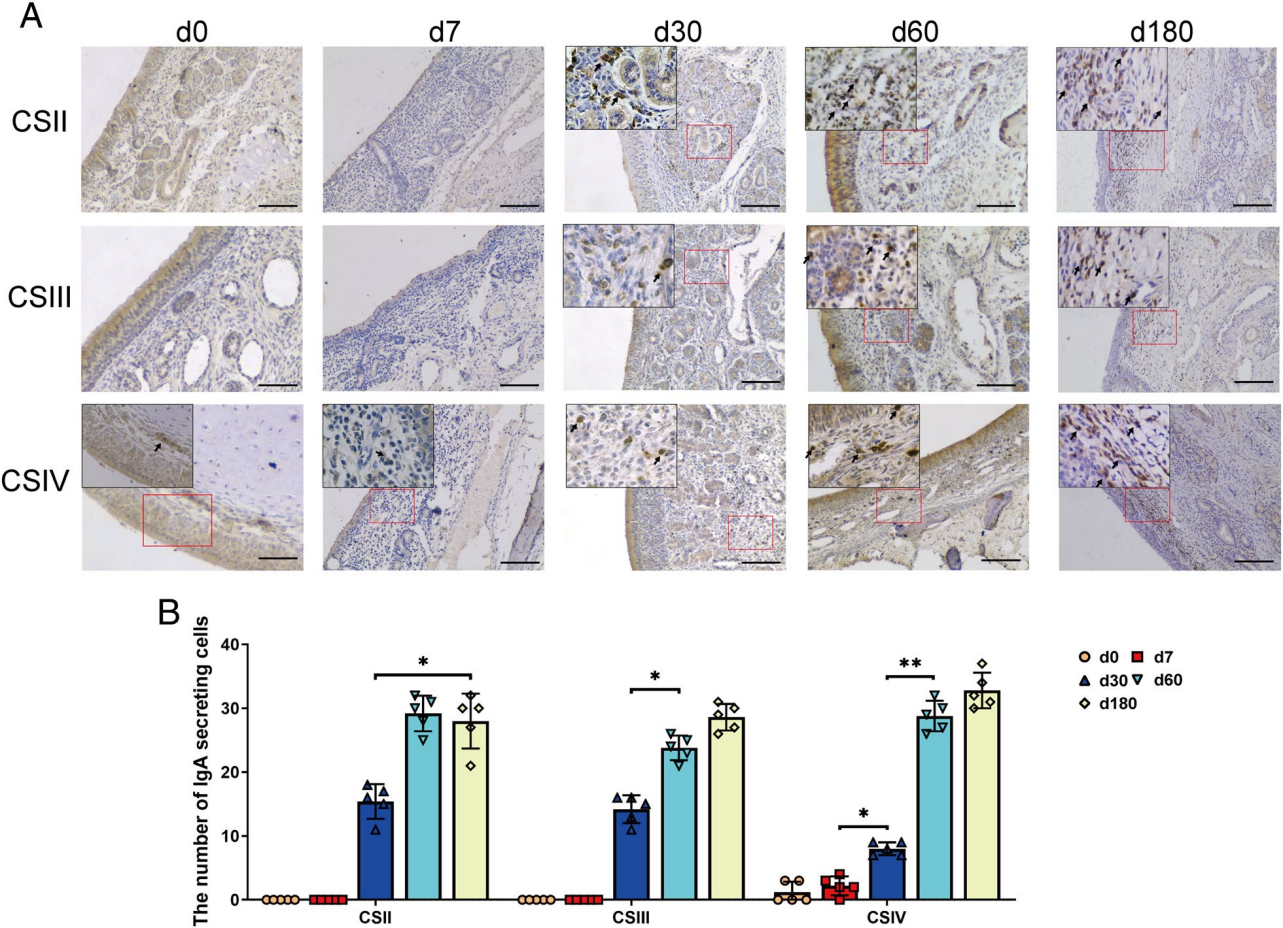
**The location of IgA**^**+**^** B cells within the nasal epithelium of pigs at different growth stages.** **A** IHC staining for IgA^+^ B cells in the nasal epithelium. B-cell-specific IgA protein was stained brown. CSII represented the anterior part of the respiratory region, CSIII represented the rear part of the respiratory region, and CSIV represented the olfactory region. Scale bars: 100 μm. **B** Quantification analysis of IgA^+^ B cells. The number of IgA-positive cells in different parts of the nasal cavity was counted in five random fields (×10) from the four cross-sections. All data shown are the mean ± SD from three independent experiments. Statistical significance was obtained using one-way ANOVA. NS no significance, *P < 0.05, **P < 0.01.

Mucosal B cells are important contributors to maintaining mucosal immunological function through the production of secretory IgA [28]. Similar to IETs, IgA^+^ cells were rarely observed in the respiratory and olfactory regions of neonatal and suckling piglets; however, they increased significantly with age (Figure 8).

**Figure 9:**
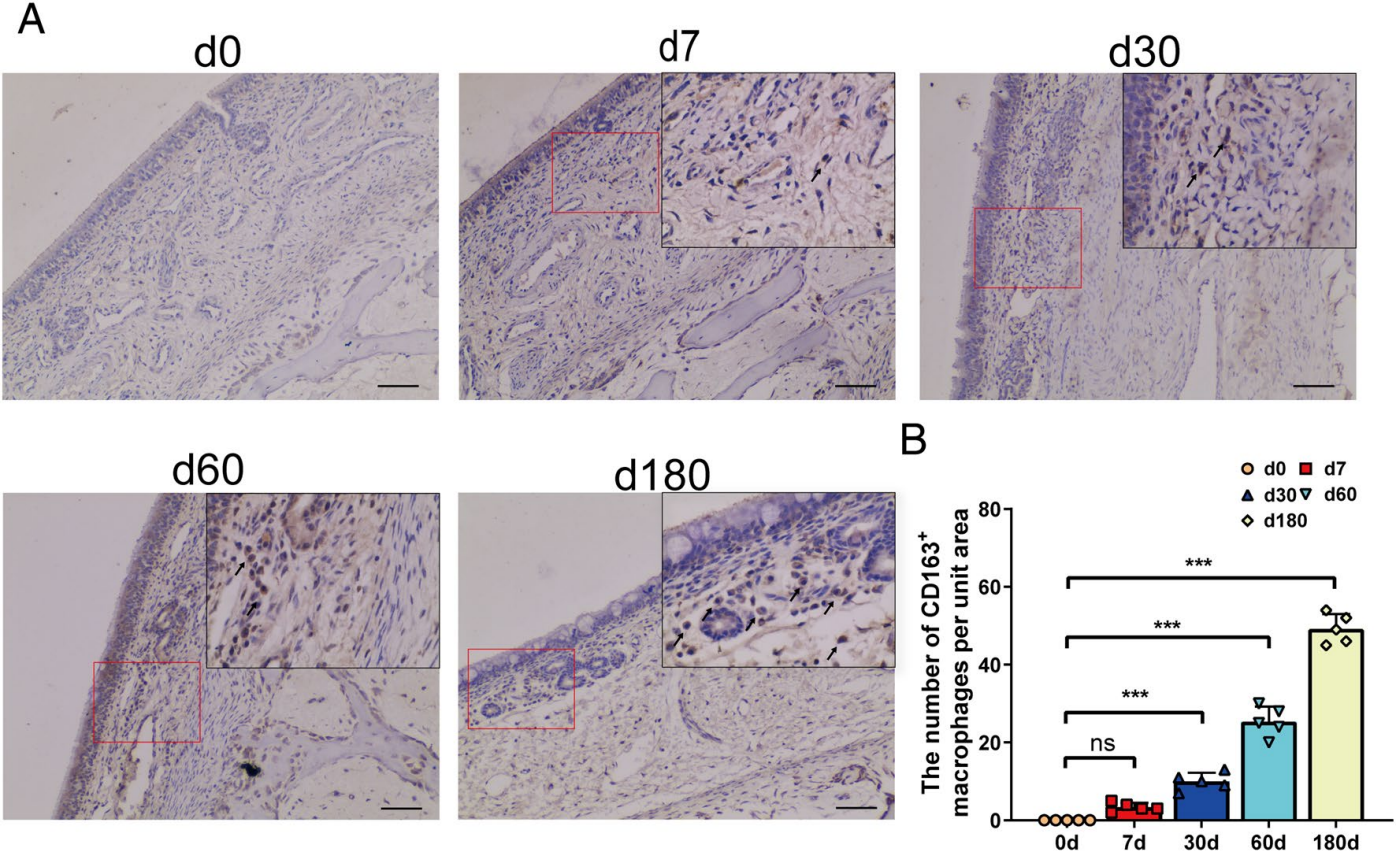
**The distribution of CD163** ^**+**^ **macrophages in the nasal mucosa of pigs at different growth stages**. **A** IHC staining for CD163^+^ macrophages in the nasal mucosa. Macrophage-specific CD163 protein was stained brown. Scale bars: 50 μm. **B** Quantification analysis of macrophages. The number of CD163^+^ macrophages in the rear part of the respiratory region from pigs at different growth stages was counted in five random fields (×10) from the four cross-sections. All data shown are the mean ± SD from three independent experiments. Statistical significance was obtained using one-way ANOVA. NS no significance, ***P < 0.001.

Macrophages are key antigen-presenting cells that bridge innate and adaptive immunity and play a pivotal role in maintaining mucosal immune homeostasis. IHC results revealed that only a few macrophages were present in the nasal respiratory region of suckling and weaning piglets, and the number of CD163^+^ macrophages increased gradually with age. Furthermore, a significant increase in the number of mucosal macrophages was observed from the fattening to the finishing stage (Figure 9).

### Microbial barrier characteristics of the nasal cavity at different growth stages

After stringent quality control, 25 nasal samples yielded 541 183 sequences, with a mean of 21 647 per sample (range, 11 080–35 688). The sequences were clustered into 22 320 OTUs, and each sample contained a mean of 892 OTUs (range, 506–1299 OTUs). A Venn diagram displayed 252 OTUs shared among the five age groups, and each group had unique OTUs (Additional file 6). There were 585 unique OTUs in neonatal piglets, 62 unique OTUs in suckling piglets, 207 unique OTUs in weaning pigs, 98 unique OTUs in fattening pigs, and 203 unique OTUs in finishing pigs. The following three alpha diversity indices were used to evaluate modifications in microbial alpha diversity: the Sobs (observed OTUs) diversity index, the Shannon diversity index, and the Chao1 index (Additional file 6). The three diversity estimators exhibited the same trend, with changes in alpha diversity observed with increasing age. The alpha diversity results indicated that the microbial diversity and richness in the nasal cavity decreased from birth to the suckling stage; then, they gradually increased and peaked in the finishing phase. To further examine the impact of age on microbial beta diversity, a principal coordinate analysis of the OTU diversity data was performed (Additional file 6). The principal coordinate analysis plot suggested that the microbial compositions at days 7, 30, and 60 formed clusters with partial overlap, although marked differences were observed in the microbiomes of each group.

**Figure 10:**
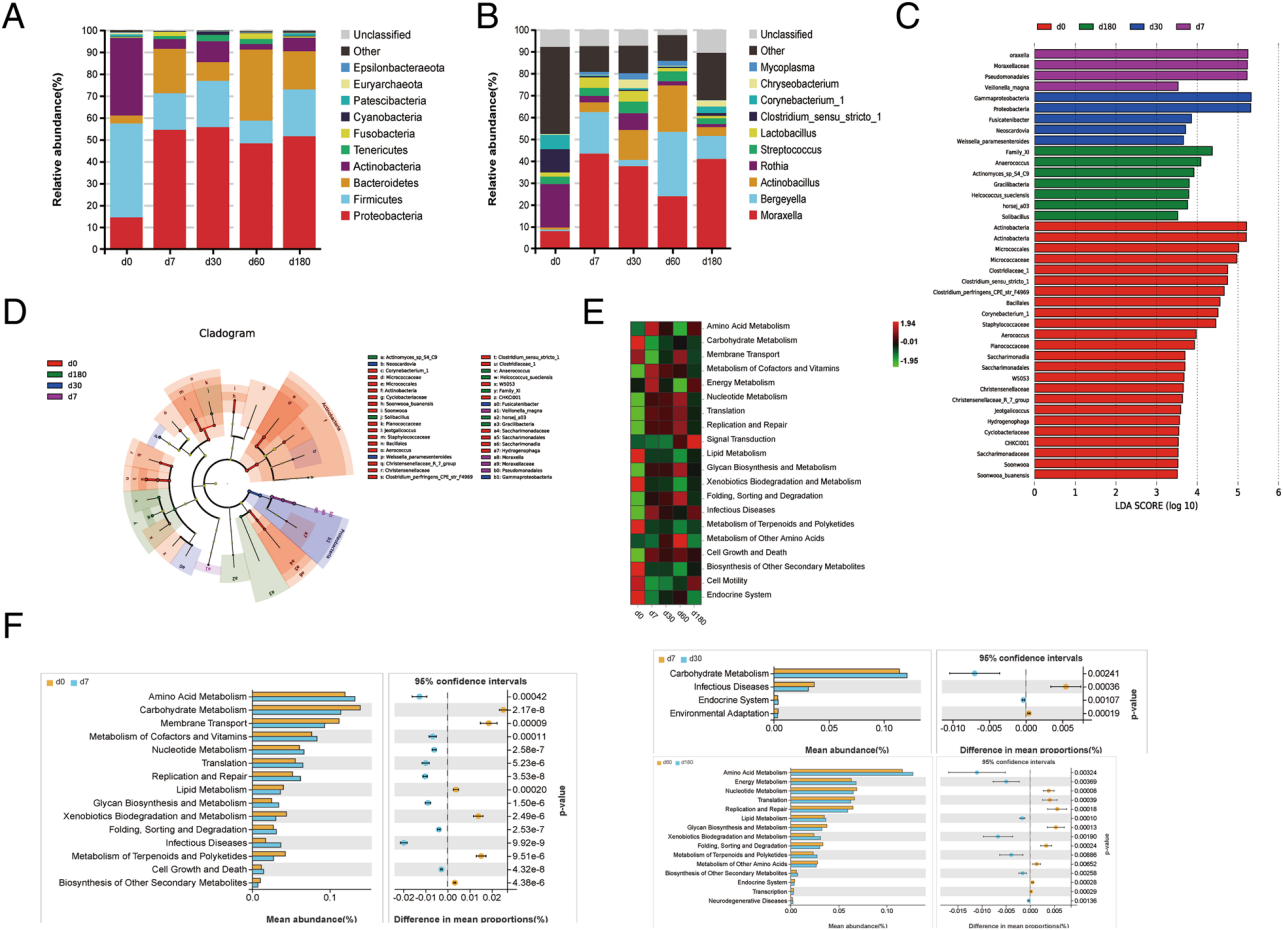
**Taxonomic analysis of pig nasal cavity microorganisms at different ages.** Relative abundance of bacterial phyla (**A**) and genera (**B**) in the nasal cavity of pigs at different ages. The top 8 classes were described for each period, and all other classes were grouped as “Other” or “Unclassified.” LEfSe analysis based on OTUs characterizes microbiomes in the porcine nasal cavity at different ages. **C** Indicator bacteria with linear discriminant analysis (LDA) scores of 3.5 or greater are displayed. **D** Cladogram representing the LEfSe results of which taxa (highlighted by small circles and shading) are enriched in the nasal microbiome at different ages. **E**, **F** The microbial community function from all age groups was predicted using Tax4Fun. **E** Heatmap of representative microbial functions among the groups. **F** Comparison of significant differences in microbial functions between the different age groups. *P* values were calculated with Welch’s t test.

The OTUs were classified into 31 bacterial phyla and 585 genera. At the phylum level, the total abundances of *Firmicutes*, *Bacteroidetes*, *Proteobacteria*, and *Actinobacteria* accounted for more than 90% of all nasal bacteria throughout the growth stages (Figure 10A). The relative abundance of the bacterial phyla in the nasal microbiota was markedly altered with increasing age. On the day of birth, the nasal region had high abundances of the phyla *Firmicutes* (42.9%) and *Actinobacteria* (35.4%) and relatively lower abundances of the phyla *Proteobacteria* (14.4%) and *Bacteroidetes* (3.6%). From the day of birth to 7 days of age, we observed a significant decrease in the abundance of *Firmicutes* (16.8%) and *Actinobacteria* (4.5%) and a significant increase in the abundance of bacteria (20.2%) and *Proteobacteria* (54.4%). Subsequently, the abundance of *Firmicutes* and *Actinobacteria* rapidly increased, decreased, and increased again from the suckling stage to the finishing stage; however, the abundance of *Bacteroidetes* exhibited the opposite trend (decrease-increase–decrease). The abundance of *Actinobacteria* was < 10% among the four age groups. However, the high abundance of *Proteobacteria* (48.2%-55.6%) remained stable from the suckling stage to the finishing stage. At the genus level, five dominant bacterial groups (the abundances from high to low were as follows: *Rothia*, *Clostridium *sensu stricto* 1*, *Moraxella*, *Corynebacterium*, and *Streptococcus*) were detected in the nasal cavity of neonatal piglets; however, the trends in the other age groups were quite different (Figure 10B). The abundance of *Streptococcus spp.* remained stable throughout the growth stages. The abundances of *Rothia*, *Clostridium-*sensu*-stricto 1*, and *Corynebacterium* decreased significantly during the suckling stage and remained at relatively low levels during subsequent months. However, *Moraxella* continued to increase and became predominant (23.9%-43.3%) during this period, although a disruption in this trend was observed at the age of fattening. Additionally, a high abundance of *Bergeyella* was observed at the suckling and fattening stages, and a low abundance of mycoplasma was detected from the suckling stage to the fattening stage.

A linear discriminant analysis of the effect size was performed to corroborate the representative taxa further and screen the differential microbes among the groups. Using the linear discriminant analysis, 40 discriminative biomarkers with scores greater than 3.5 were observed (24 for neonatal pigs, four for suckling pigs, five for weaning pigs, and seven for finishing pigs) (Figure 10C). There were no biomarkers identified in fattening pigs. Neonatal pigs had a unique microbiota because of the high amounts of the genera *Clostridium-*sensu*-stricto-1* and *Christensenellaceae-R-7* (both classes within *Clostridia*), *Corynebacterium-1* (class within *Actinobacteria*), *Soonwooa* (class within *Bacteroidia*), and *Jeotgalicoccus* and *Aerococcus* (both classes within *Bacilli*). However, significant changes in the dominant phylotypes, *Moraxellacea* (class within *Gammaproteobacteria*) and *Veillonella* (class within *Negativicutes*), were detected in suckling piglets. After weaning, the nasal mucosa was more highly colonized by *Neoscardovia* (class within *Actinobacteria*) and *Fusicatenibacter* (class within *Clostridia*); this was different from the observations at birth and at the weaning stage. The genera *Helcococcus* and Family-XI (class within *Clostridia*) and horsej-a03 (class within *Oligosphaeria*) were the dominant phylotypes in the nasal microbiota of the finishing pigs.

Furthermore, the functional profiles of nasal microbial communities of all age groups were predicted using Tax4Fun. The heatmap shows the changes in specific functional pathways among each group (Figure 10E). The activity levels of the most predicted metabolic pathways (including carbohydrate metabolism, lipid metabolism, xenobiotic biodegradation and metabolism, metabolism of terpenoids and polyketides, and biosynthesis of other secondary metabolites) were the highest soon after birth and decreased with age. However, some predicted pathways (including infectious diseases, cell growth, death, and signal transduction) showed the opposite trend; the activity levels were the lowest at birth and gradually increased during the first month of life. Additionally, the expression of proteins in some enriched pathways (especially translation, replication, repair, and nucleotide metabolism) increased from birth to fattening, but they decreased during the finishing stage. Welch’s t test was used to test for significant differences in the activities of the pathways among the groups (Figure 10F). Compared to those in neonatal piglets, some of the predicted metabolism pathways of the suckling piglets were significantly inhibited, especially xenobiotic biodegradation and metabolism, biosynthesis of other secondary metabolites, and metabolism of terpenoids and polyketides. In contrast, the infection disease pathway was activated. Only a few of the predicted pathways were affected after weaning. However, most of the predicted pathways enriched in fattening pigs were significantly different from those of finishing pigs, including multiple pathways involved in metabolism, genetic information processing, human diseases, and organismal systems.

## Discussion

To increase knowledge of the nasal cavity of commercial pigs, our study explored the structural and functional characteristics at different growth stages. Histological analysis revealed that the epithelial thickness of the nasal mucosa and the number of capillaries in the lamina propria increased with age. This suggests that the nasal mucosa undergoes numerous structural and functional changes during pig growth and development. In the vestibular region, the junction between the basal layer of the epithelium and connective tissue becomes more apparent with age. Particularly in finishing pigs, rete pegs characterized by inwards projections of the epidermis into the connective tissue could be observed. The formation of these structures not only ensures an epithelial nutrient supply through increased contact area but also enhances the protection and frictional resistance of the epithelium. The structural complexity of the respiratory and olfactory regions also increased with age, and the enlarged spiral structure of the middle and inferior turbinates enhanced the interface between the nasal mucosa and the external environment (Additional file 7). These regions were covered by a pseudostratified epithelial layer and therefore comprised the primary gateways for invading pathogens in the nasal cavity. Our previous studies on Bama minipigs showed that some lymphoid follicles are distributed randomly in these regions, whereas the lymphoid tissue is barely visible in commercial fattening pigs. This may be an important reason for the weaker disease resistance of commercial pigs compared to Bama minipigs [29]. Moreover, the age-dependent increases in the number of glands and mucus-secreting cells in the nasal mucosa indicated the continuously improved secretory function of the nasal cavity during pig growth.

An investigation of the nasal epithelial barrier showed high expression of tight junction proteins after birth that decreased significantly during the suckling stage but increased again during the weaning stage. A similar trend with age was observed in the proliferative capacity of nasal epithelial cells. The developmental features of the nasal epithelium were quite different from those previously reported for the porcine intestinal epithelial barrier, which displayed increased tight junction protein expression and proliferative capacity after birth that decreased dramatically after weaning [30, 31]. These results indicate the independence of epithelial development in different tissues and organs of pigs; they also suggest that weaning stress may have less impact on the nasal epithelial barrier than on the intestinal epithelial barrier, although it has been reported to severely influence the physiological functions of piglets [32]. Diminished epithelial barrier function in the nasal mucosa may decrease the resistance of suckling piglets to respiratory pathogen infection and therefore warrants further attention. Moreover, the proliferative ability of nasal epithelial cells decreased during the fattening/finishing period, which is consistent with a previous study reporting a decrease in the proliferative ability of epithelial cells in adult mammals with increasing age [33].

Regarding the nasal immune barrier, our study showed that most of the pattern recognition receptors in the nasal mucosa exhibited low expression in neonatal piglets and that only a few innate immune cells were distributed in the lamina propria. These observations suggest an incomplete nasal immunological barrier in neonatal piglets. The expression of TLR2 and TLR4 in the nasal mucosa significantly increased during the suckling stage. Because TLR2 and TLR4 are mainly involved in the pattern recognition of pathogenic bacteria [34], their activation in the nasal mucosa may be associated with invading conditional pathogenic bacteria, which may have derived from the udder skin of the lactating sows [35]. The expression of TLRs and the number of innate immune cells in the nasal mucosa significantly increased from the weaning to the finishing stage, which could be a critical period in developing the nasal immunological barrier. During the fattening period, although most pattern recognition receptors were expressed at high levels, TLR3 and TLR4 were significantly decreased. These findings are consistent with a previous study showing that the expression level of TLR3 decreased with increasing age in the porcine intestinal mucosa [31]. The RIG-like receptors RIG-1 and MDA5 exhibited opposite expression trends in the nasal mucosa during the growth of pigs. The expression of RIG-1 was high after birth, significantly decreased thereafter, and was maintained at a low level until the finishing stage. The expression of MDA5 was low at birth but increased significantly during the finishing stage. The opposite expression trends for RIG-1 and MDA5 were observed in the mouse gut, although the regulatory mechanisms involved require further investigation [36]. Because the pattern recognition receptors TLR3 and RIG-I are critical players in the innate antiviral response [37], decreased expression of both may lead to high susceptibility to porcine reproductive and respiratory syndrome virus, porcine pseudorabies virus, swine influenza virus, and other porcine respiratory viruses in finishing pigs.

The commensal microbiota forms a natural barrier on the surface of the nasal mucosa and participates in regulating and maintaining the integrity of the mucosal mechanical barrier and immune function [38]. The community structure of the nasal commensal microbiota is established during infancy and changes with age [39]. In our study, *Firmicutes*, *Actinobacteria*, *Proteobacteria*, and *Bacteroidetes* comprised the dominant phyla in the nasal cavities of neonatal piglets. This finding is consistent with previous studies investigating infants’ nasal microbiota and other commercial pig breeds [39, 40]. A dramatic decrease in the relative abundance of the phyla *Firmicutes* and *Actinobacteria* was observed from birth to the suckling stage.

In contrast, the abundance of *Bacteroidetes* and *Proteobacteria* increased during the same period. The phyla *Bacteroidetes* and *Proteobacteria* are known to include multiple potentially pathogenic bacteria, and members of these taxa have pro-inflammatory properties [41, 42]. There were also notable increases in *Bergeyella* and *Moraxella* observed during the present study.

The nasal microbial taxon diversity was significantly reduced during the suckling stage. Increased *Moraxella* in the nasal cavity may have contributed to this phenomenon [43]. From the weaning to the finishing stage, the relative abundance of the Proteobacteria phylum remained stable, but the phyla *Firmicutes*, *Bacteroidetes*, and *Actinobacteria* changed dynamically with pig ageing. In finishing pigs, *Proteobacteria*, *Bacteroidetes*, and *Firmicutes* were identified as the core phyla of nasal microbiota; among these, *Actinobacter*, *Moraxella*, and *Bergerella*, which were the three dominant genera, may become opportunistic pathogens that could cause respiratory infections in finishing pigs.

Functional analysis of the nasal microbiota revealed that the function of metabolic signalling pathways was significantly increased at birth, which may be related to the top biomarker *Clostridium-*sensu*-stricto 1* in the nasal cavity of neonatal piglets. These bacteria are involved in metabolizing carbohydrates, lipids, and other substances in the body [44]. Furthermore, during the suckling stage, the significant upregulation of infectious disease function may be related to the top biomarker, *Moraxella*, which again indicates a risk of respiratory infection in suckling piglets. Moreover, the rapid weight gain of pigs during the weaning stage may be associated with the top biomarker, *Fusicatenibacter*, which plays a critical role in modulating host carbohydrate and lipid metabolism [45].

In summary, our study revealed the compositional characteristics of the nasal mechanical, immunological, and biological barriers, constituting the nasal mucosa’s essential defence during commercial pig growth. These findings not only improve our understanding of the histological characteristics of the nasal cavity of commercial pigs but also provide a theoretical basis for future research on nasal mucosal immunity. Of note, reduced proliferation and tight junction protein expression of the nasal epithelium, a dramatic decrease in microbial diversity and an increase in potentially pathogenic bacteria in the nasal cavity were detected in suckling piglets, suggesting a high risk of respiratory pathogen infection during this period.

## Supplementary Information

**Additional file 1. Primers used for real-time PCR.**

**Additional file 2. HE staining of the vestibular region of the nasal cavity in different growth stages.** (A) Diagrams of pig nasal cavity cross-Section I (corresponding to the vestibular region). (B-F) Representative images of HE-stained nasal vestibular regions from pigs at different ages, including 0 days old (B), 7 days old (C), 30 days old (D), 60 days old (E), and 180 days old (F). The red frame in each figure indicates the inferior nasal concha (a) and nasal septum (b); magnified images of the corresponding region are shown on the right of the figure. Scale bars: (B-F) 2 mm; (a, b) 50 μm.

**Additional file 3. Histological analysis of the four cross-sections of the porcine nasal cavity.** (A) Quantitative analysis of the epithelial thickness of the four regions of the porcine nasal cavity. The epithelial thickness of the nasal mucosa was measured using ImageJ software, and five visual fields (40 ×) were randomly selected from the five individual sections. All the measurements summarized in the column plot are provided as the average epithelial thickness per group. (B) Quantitative analysis of the number of glands in the lamina propria of the four regions of the porcine nasal cavity. The number of glands was counted in five randomly selected visual fields (10 ×) of the five individual sections, summarized in a column plot. (C) Quantitative analysis of the number of capillaries in the four regions of the nasal cavity. The number of capillaries in the nasal mucosa was counted in five randomly selected visual fields (10 ×) of the five individual sections. All data shown are the mean ± SD from three independent experiments. Statistical significance was obtained using one-way ANOVA. The differences are indicated by different letters. Letters above the graphs indicate statistical significance in which treatments with a letter in common are not significantly different from each other.

**Additional file 4. The distribution of lymphoid follicles in the nasal respiratory region of pigs at different ages.** Representative images of HE-stained nasal respiratory regions from pigs at different ages, including 7 days old (A), 60 days old (B) and 180 days old (C). The red frame in each figure indicates the different parts of the inferior nasal concha (a and b), and magnified images of the corresponding region are shown on the right of the figure. Black asterisks mark the lymphoid follicle. CSII: the anterior part of the respiratory region; CSIII: the rear part of the respiratory region; Scale bars: (A to C) 2 mm; (a, b) 200 μm.

**Additional file 5. HE staining of the olfactory region of the nasal cavity in different growth stages.** (A) Diagrams of pig nasal cavity cross-section IV (corresponding to the olfactory region). (B-F) Representative images of HE-stained nasal olfactory regions from pigs at different ages, including 0 days old (B), 7 days old (C), 30 days old (D), 60 days old (E), and 180 days old (F). The red frame in each figure indicates the middle nasal concha (a) and nasal septum (b); magnified images of the corresponding region are shown on the right of the figure. Scale bars: (B-F) 2 mm; (a, b) 50 μm.

**Additional file 6. OTU-based community composition and diversity analysis.** (A) Venn diagram showing the shared and unique OTUs in nasal swab samples collected from different age groups. Based on the OTU composition, the biodiversity of the samples (alpha diversity) from the different age groups was calculated with the Shannon (B), Sob (C) and Chao1 (D) indices. The beta diversity of bacterial communities from the different age groups is shown in the PCoA plot.

**Additional file 7. Quantitative analysis of the total area of the nasal concha in the nasal respiratory and olfactoria regions.** The area of the nasal concha was measured using ImageJ software, and the results were obtained from five individual sections. The histogram shows the mean ± SD for the area of middle or inferior turbinates of the nasal cavity at different growth stages. Data are representative of three independent experiments. The differences are indicated by different letters. Letters above the graphs indicate statistical significance in which treatments with a letter in common are not significantly different from each other.

## Data Availability

The data that support the findings of this study are available upon request to the corresponding authors.
