# Supplementary material for: Characteristics of the nasal mucosa of commercial pigs during normal development
Source: Vet Res. 2023 Apr 24;54:37. doi: 10.1186/s13567-023-01164-y (PMC10123470; doi:10.1186/s13567-023-01164-y)
Supplement: Supplementary file 1 — Additional file 1. Primers used for real-time PCR. [file 13567_2023_1164_MOESM1_ESM.docx]

**Additional file 1 Primer sequences used for RT-qPCR**

| Gene Primers | Sequence (5–3) | Orientation |
| --- | --- | --- |
| TLR1 | AGATTTCGTGCCACCCTATG  CCTGGGGGATAAACAATGTG | Forward  Reverse |
| TLR2 | GAGTCTGCCACAACTCAAAGA  CAGAACTGACAACATGGGTAGAA | Forward  Reverse |
| TLR3 | GAGCAGGAGTTTGCCTTGTC  GGAGGTCATCGGGTATTTGA | Forward  Reverse |
| TLR4 | TCATCCAGGAAGGTTTCCAC  TGTCCTCCCACTCCAGGTAG | Forward  Reverse |
| TLR5 | GGTCCCTGCCTCAGTATCAA  GTTGAGAAACCAGCTTGACG | Forward  Reverse |
| TLR6 | TCAAGCATTTGGACCTCTCA  TTCCAAATCCAGAAGGATGC | Forward  Reverse |
| TLR7 | TCTGCCCTGTGATGTCAGTC GCTGGTTTCCATCCAGGTAA | Forward  Reverse |
| TLR8 | CTGGGATGCTTGGTTCATCT CATGAGGTTGTCGATGATGG | Forward  Reverse |
| TLR9 | AGGGAGACCTCTATCTCCGC  AAGTCCAGGGTTTCCAGCTT | Forward  Reverse |
| TLR10 | GCCCAAGGATAGGCGTAAAT  CTCGAGACCCTTCATTCAGC | Forward  Reverse |
| Mda5 | CAAGCTTGGGGAACGATGATG  TAGCTGGTGATGGGGTCCTC | Forward  Reverse |
| RIG-I | GAGCCCTTGTGGATGCTTTA GGGTCATCCCTATGTTCTGATTC | Forward  Reverse |
