# Supplementary figures and images for: Characteristics of the nasal mucosa of commercial pigs during normal development
Source: Vet Res. 2023 Apr 24;54:37. doi: 10.1186/s13567-023-01164-y (PMC10123470; doi:10.1186/s13567-023-01164-y)

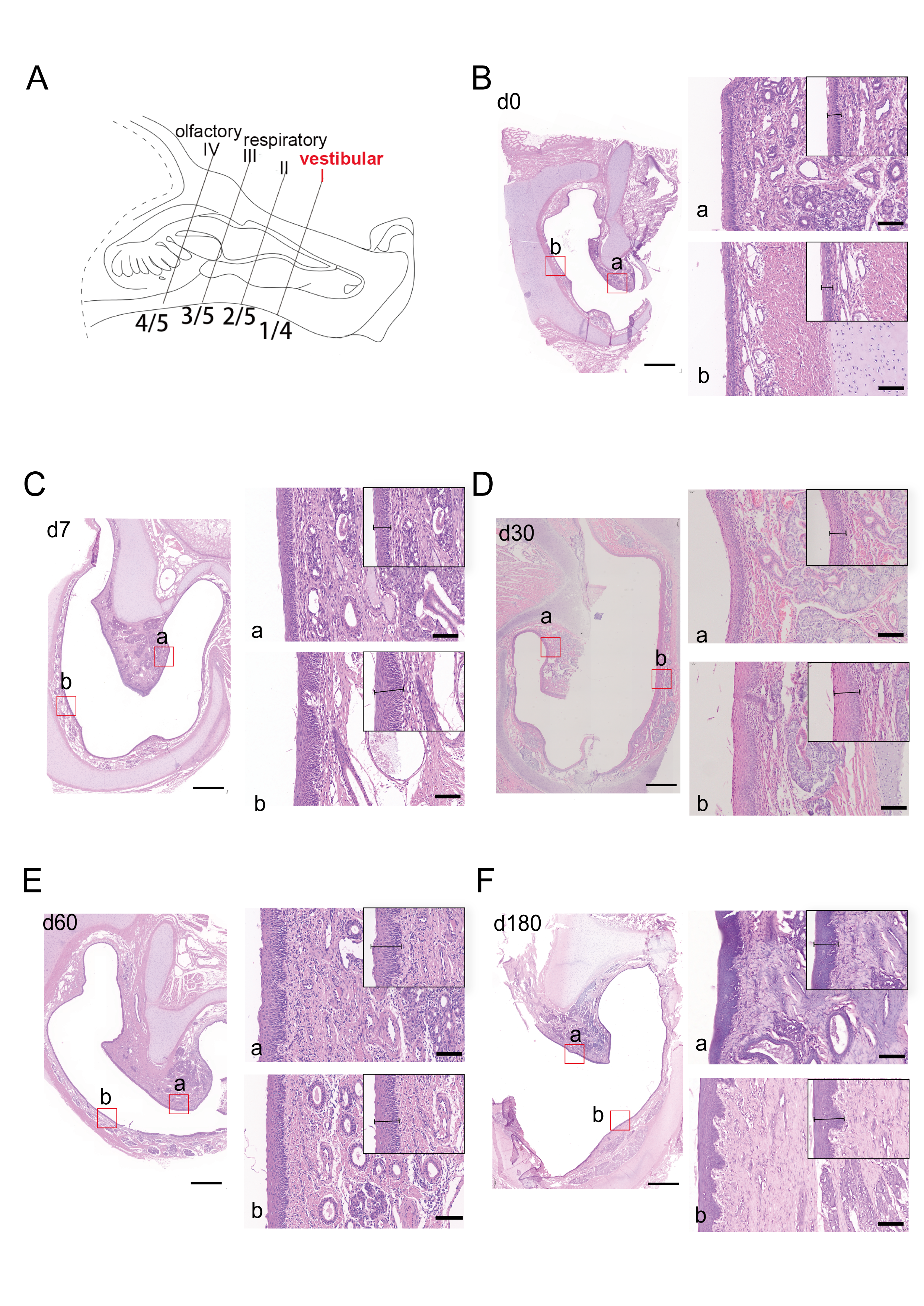

Supplement: Supplementary file 2 — Additional file 2. HE staining of the vestibular region of the nasal cavity in different growth stages. (A) Diagrams of pig nasal cavity cross-Section I (corresponding to the vestibular region). (B-F) Representative images of HE-stained nasal vestibular regions from pigs at different ages, including 0 days old (B), 7 days old (C), 30 days old (D), 60 days old (E), and 180 days old (F). The red frame in each figure indicates the inferior nasal concha (a) and nasal septum (b); magnified images of the corresponding region are shown on the right of the figure. Scale bars: (B-F) 2 mm; (a, b) 50 μm. [file 13567_2023_1164_MOESM2_ESM.tif]

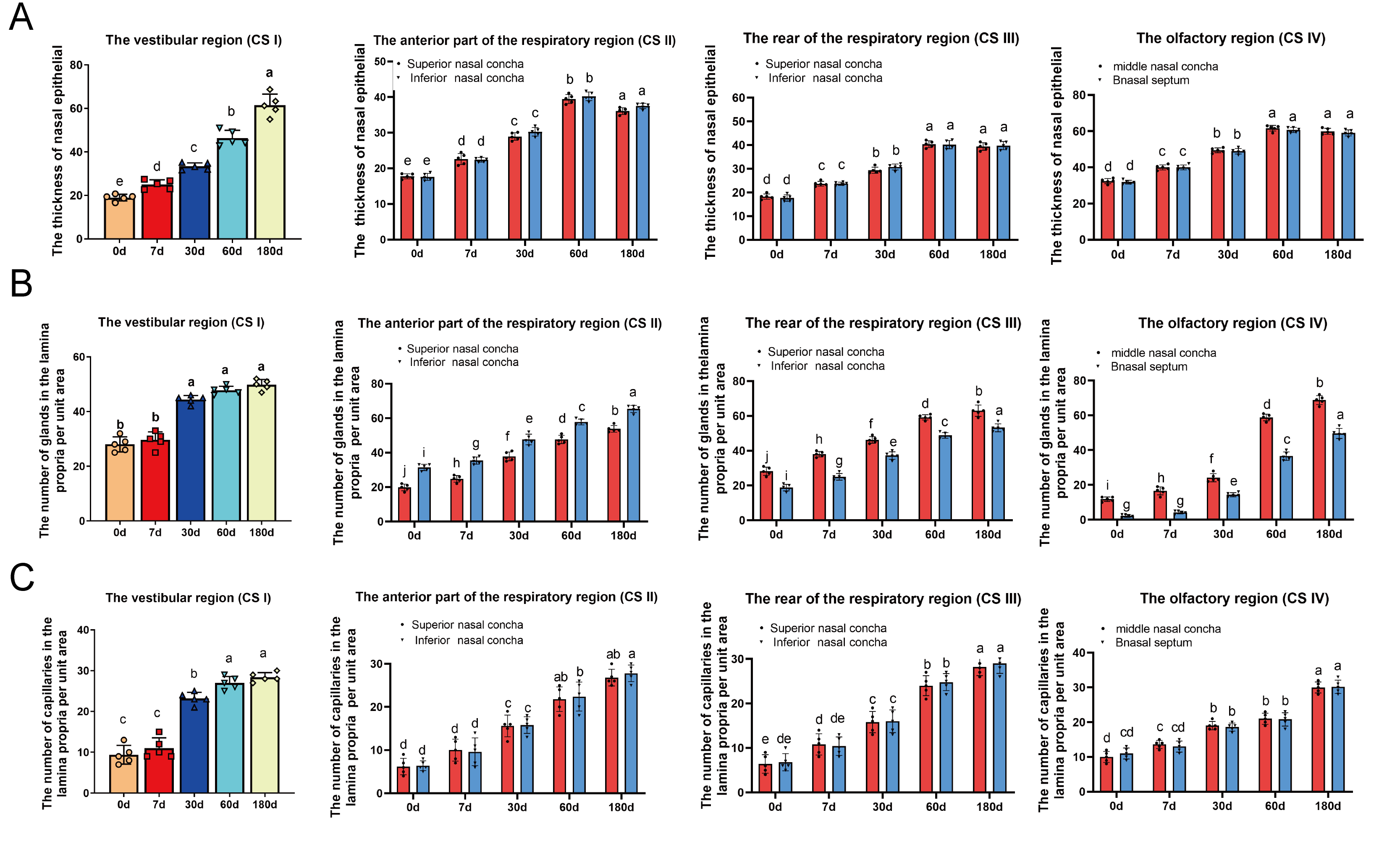

Supplement: Supplementary file 3 — Additional file 3. Histological analysis of the four cross-sections of the porcine nasal cavity. (A) Quantitative analysis of the epithelial thickness of the four regions of the porcine nasal cavity. The epithelial thickness of the nasal mucosa was measured using ImageJ software, and five visual fields (40 ×) were randomly selected from the five individual sections. All the measurements summarized in the column plot are provided as the average epithelial thickness per group. (B) Quantitative analysis of the number of glands in the lamina propria of the four regions of the porcine nasal cavity. The number of glands was counted in five randomly selected visual fields (10 ×) of the five individual sections, summarized in a column plot. (C) Quantitative analysis of the number of capillaries in the four regions of the nasal cavity. The number of capillaries in the nasal mucosa was counted in five randomly selected visual fields (10 ×) of the five individual sections. All data shown are the mean ± SD from three independent experiments. Statistical significance was obtained using one-way ANOVA. The differences are indicated by different letters. Letters above the graphs indicate statistical significance in which treatments with a letter in common are not significantly different from each other. [file 13567_2023_1164_MOESM3_ESM.tif]

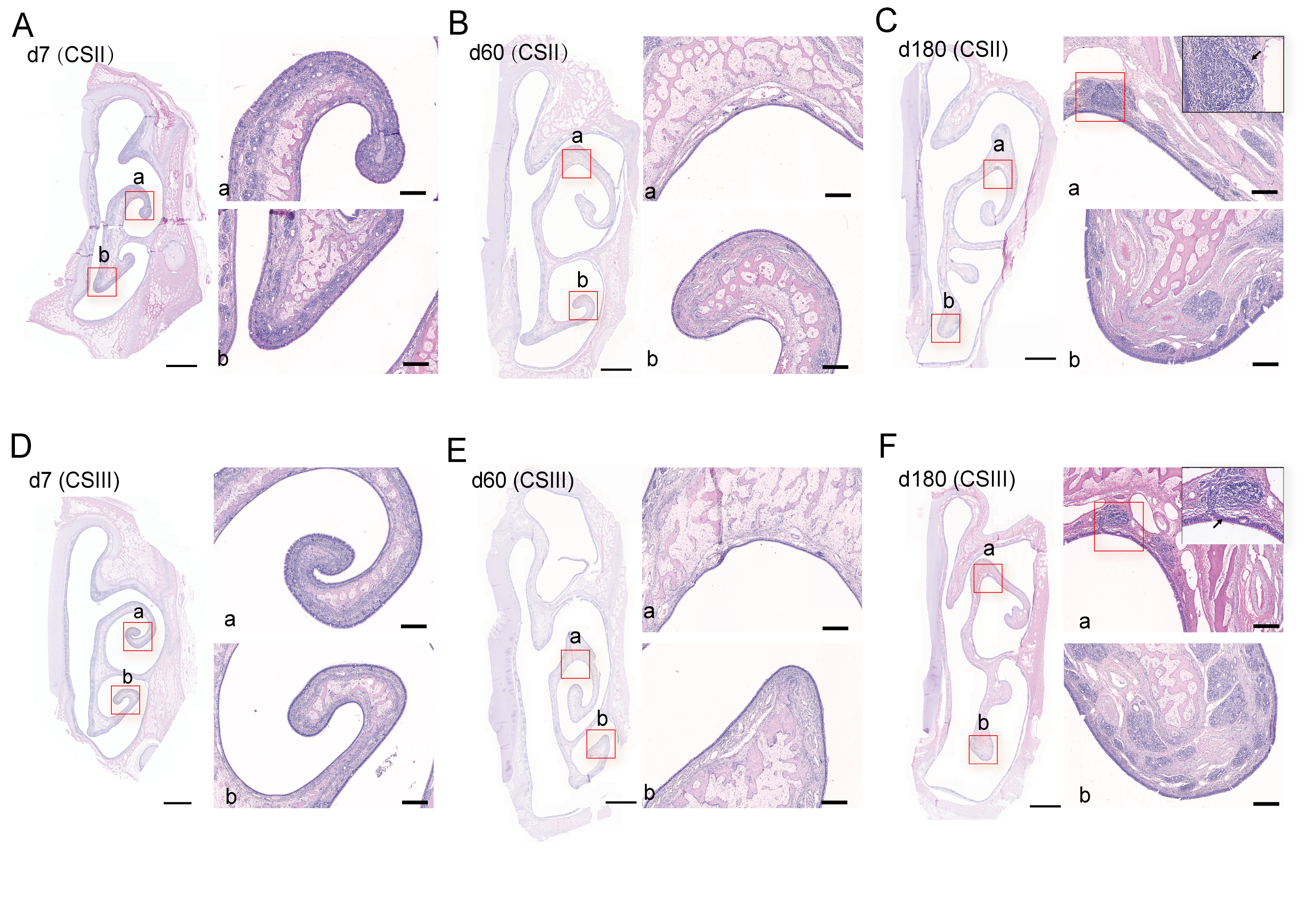

Supplement: Supplementary file 4 — Additional file 4. The distribution of lymphoid follicles in the nasal respiratory region of pigs at different ages. Representative images of HE-stained nasal respiratory regions from pigs at different ages, including 7 days old (A), 60 days old (B) and 180 days old (C). The red frame in each figure indicates the different parts of the inferior nasal concha (a and b), and magnified images of the corresponding region are shown on the right of the figure. Black asterisks mark the lymphoid follicle. CSII: the anterior part of the respiratory region; CSIII: the rear part of the respiratory region; Scale bars: (A to C) 2 mm; (a, b) 200 μm. [file 13567_2023_1164_MOESM4_ESM.tif]

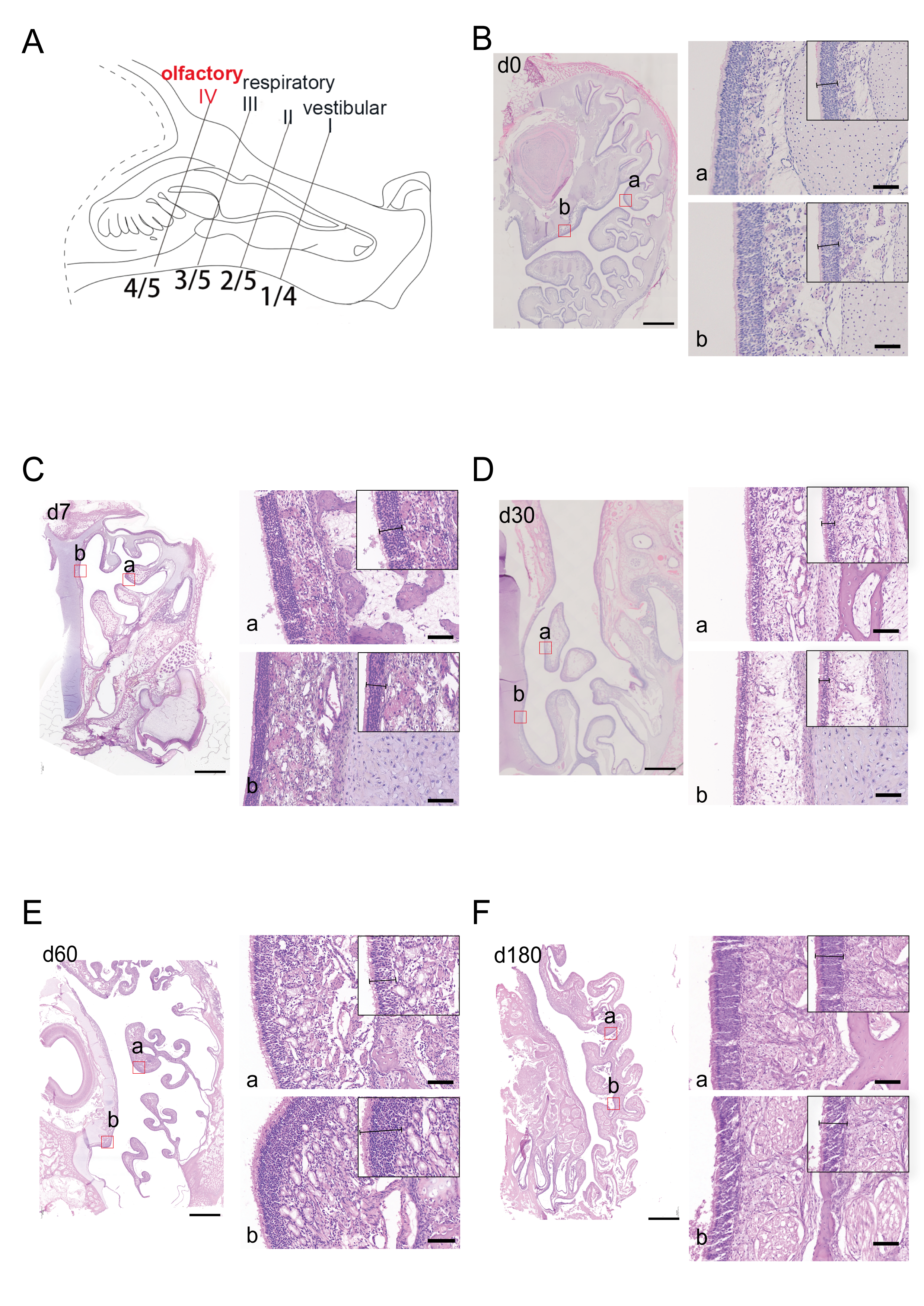

Supplement: Supplementary file 5 — Additional file 5. HE staining of the olfactory region of the nasal cavity in different growth stages. (A) Diagrams of pig nasal cavity cross-section IV (corresponding to the olfactory region). (B-F) Representative images of HE-stained nasal olfactory regions from pigs at different ages, including 0 days old (B), 7 days old (C), 30 days old (D), 60 days old (E), and 180 days old (F). The red frame in each figure indicates the middle nasal concha (a) and nasal septum (b); magnified images of the corresponding region are shown on the right of the figure. Scale bars: (B-F) 2 mm; (a, b) 50 μm. [file 13567_2023_1164_MOESM5_ESM.tif]

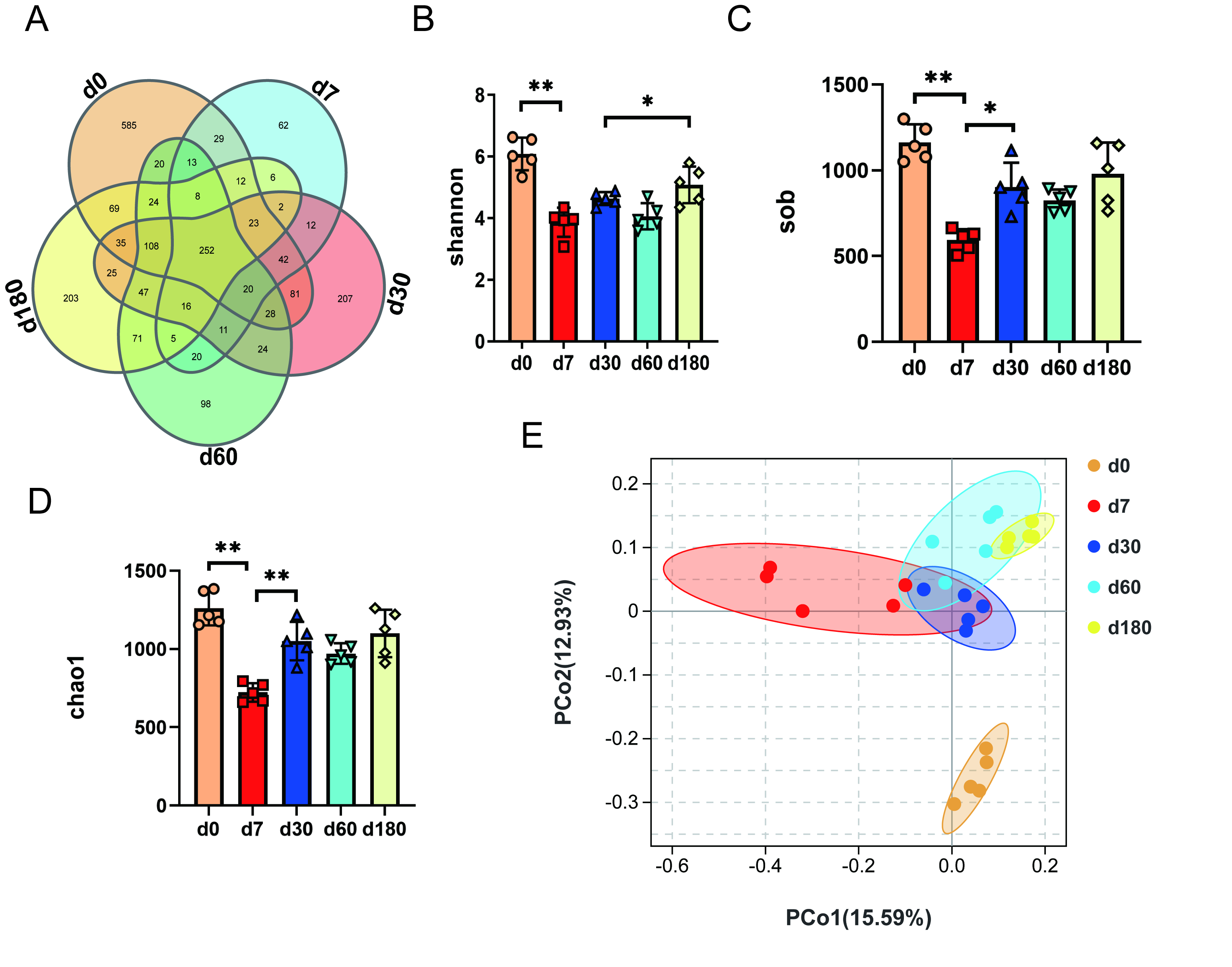

Supplement: Supplementary file 6 — Additional file 6. OTU-based community composition and diversity analysis. (A) Venn diagram showing the shared and unique OTUs in nasal swab samples collected from different age groups. Based on the OTU composition, the biodiversity of the samples (alpha diversity) from the different age groups was calculated with the Shannon (B), Sob (C) and Chao1 (D) indices. The beta diversity of bacterial communities from the different age groups is shown in the PCoA plot. [file 13567_2023_1164_MOESM6_ESM.tif]

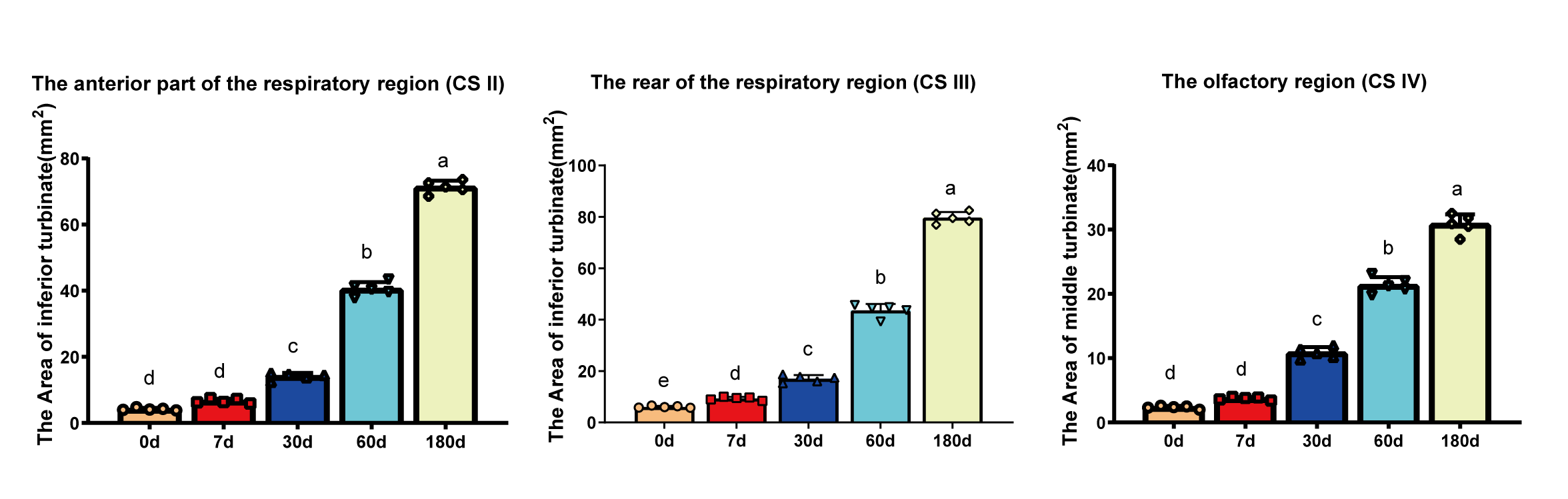

Supplement: Supplementary file 7 — Additional file 7. Quantitative analysis of the total area of the nasal concha in the nasal respiratory and olfactoria regions. The area of the nasal concha was measured using ImageJ software, and the results were obtained from five individual sections. The histogram shows the mean ± SD for the area of middle or inferior turbinates of the nasal cavity at different growth stages. Data are representative of three independent experiments. The differences are indicated by different letters. Letters above the graphs indicate statistical significance in which treatments with a letter in common are not significantly different from each other. [file 13567_2023_1164_MOESM7_ESM.tif]
